# Environmental stress is the major cause of transcriptomic and proteomic changes in GM and non-GM plants

**DOI:** 10.1038/s41598-017-09646-8

**Published:** 2017-09-06

**Authors:** Rita Batista, Cátia Fonseca, Sébastien Planchon, Sónia Negrão, Jenny Renaut, M. Margarida Oliveira

**Affiliations:** 1 Departamento de Alimentação e Nutrição, Instituto Nacional de Saúde Dr. Ricardo Jorge, Av. Padre Cruz, 1649-016 Lisboa, Portugal; 2 Luxembourg Institute of Science and Technology “Environmental Research and Innovation” (ERIN) department 5, avenue des Hauts- Fourneaux, L-4362 Esch-sur-Alzette, Luxembourg; 3 Instituto de Tecnologia Química e Biológica António Xavier, Universidade Nova de Lisboa (ITQB NOVA), Av. da República, 2780-157 Oeiras, Portugal; 4 Division of Biological and Environmental Sciences and Engineering, King Abdullah University of Science and Technology (KAUST), Thuwal, 23955-6900 Saudi Arabia

## Abstract

The approval of genetically modified (GM) crops is preceded by years of intensive research to demonstrate safety to humans and environment. We recently showed that *in vitro* culture stress is the major factor influencing proteomic differences of GM *vs*. non-GM plants. This made us question the number of generations needed to erase such “memory”. We also wondered about the relevance of alterations promoted by transgenesis as compared to environment-induced ones. Here we followed three rice lines (1-control, 1-transgenic and 1-negative segregant) throughout eight generations after transgenesis combining proteomics and transcriptomics, and further analyzed their response to salinity stress on the F6 generation. Our results show that: (a) differences promoted during genetic modification are mainly short-term physiological changes, attenuating throughout generations, and (b) environmental stress may cause far more proteomic/transcriptomic alterations than transgenesis. Based on our data, we question what is really relevant in risk assessment design for GM food crops.

## Introduction

Concerns about transgenic crops’ biosafety have led governments to implement strict regulations to assess potential risks before transgenic crops are approved for commercialization^1^. Each transgenic crop regulatory approval is preceded by years of intensive research demonstrating that the crop is safe to humans, animals as well as other non-target beneficial organisms, plants and environment.

We recently demonstrated that the major factor influencing proteomic differences on transgenic *vs*. non-transgenic plants may be the *in vitro* culture stress imposed during plant transformation. We also showed that these differences were potentially memorized throughout several generations^2^. These findings lead us to question the relevance of the differences found between transgenic plants and their controls, and enlightened the potential use of negative segregants as controls for risk assessment^2^.

If, in fact, the differences found between transgenic *vs*. non-transgenic plants are mainly short-term physiological changes, they should disappear throughout generations. If so, we could wonder how many generations transgenic plants have to pass before eliminating the memory of *in vitro* culture stress. It is known that the extensive process that precedes GM plants commercialization implies their cultivation for several generations after genetic modification and before market entry. Is this period enough for total loss of potential epigenetic signals induced by genetic modification protocols?

Another question relates to the dimension of the observed differences as compared to the differences we know that often happen due to environmental stresses. In fact, previous research showed that stress induces transcriptomic and proteomic alterations in plants, and that environmental stimuli may have a crucial role in plant allergen expression^3–6^. Plant foods are mainly field cultivated and, unavoidably, subjected to diverse environmental stresses, such as salinity, temperature, water (deficit/excess), UV, pathogens etc. Although modern agriculture strategies try to minimize unwanted stimuli, still numerous factors remain unpredictable. Thus, potentially, any consumer may eat plant foods that have suffered periods of environmental stress during their growth and post-harvest treatments.

**Figure 1:**
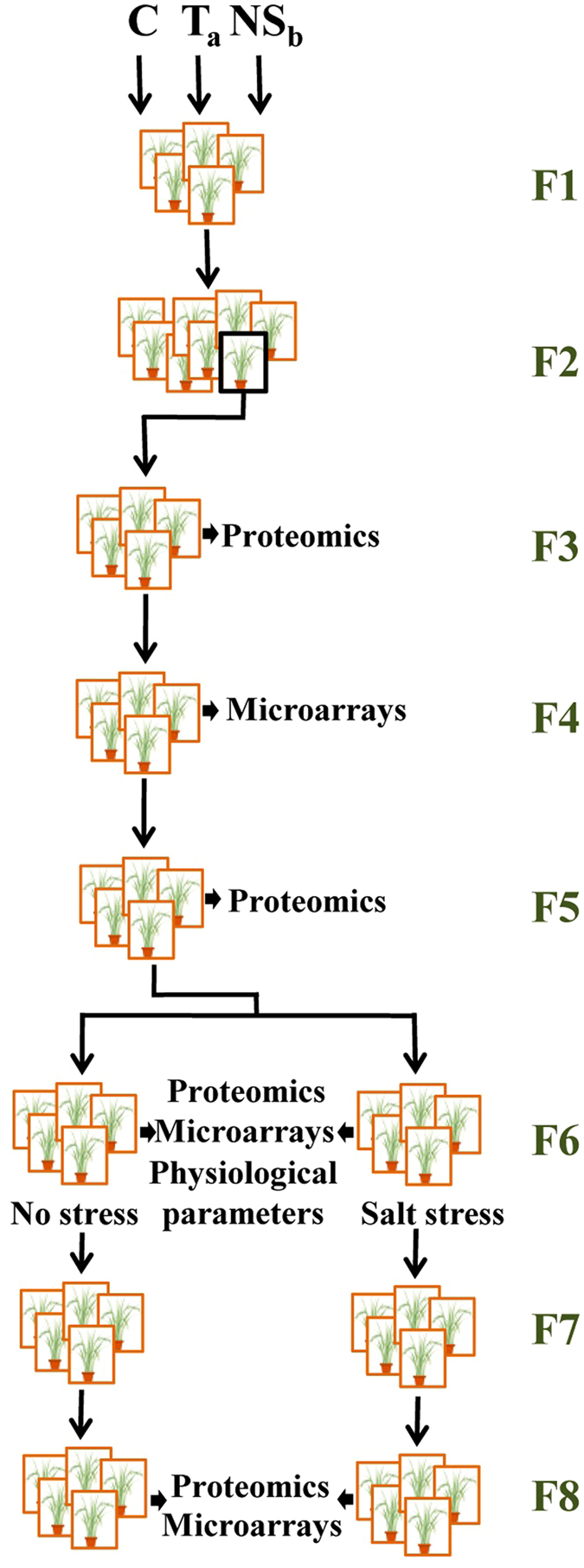
Schematic representation of the experimental strategy.

Here, we combined proteomic and microarrays analyses to compare three rice lines (a control, a transgenic and a negative segregant) from generation F3 until generation F8 after transgenesis. Our aim was to understand how *in vitro* culture-promoted alterations evolve throughout generations. We also imposed salinity stress in all lines on the F6 generation and additionally analyzed physiological parameters, to test if environmental stress-induced changes are relevant when compared with the changes that a transgenic plant may express when compared to its control (Fig. 1).

## Results and Discussion

**Figure 2:**
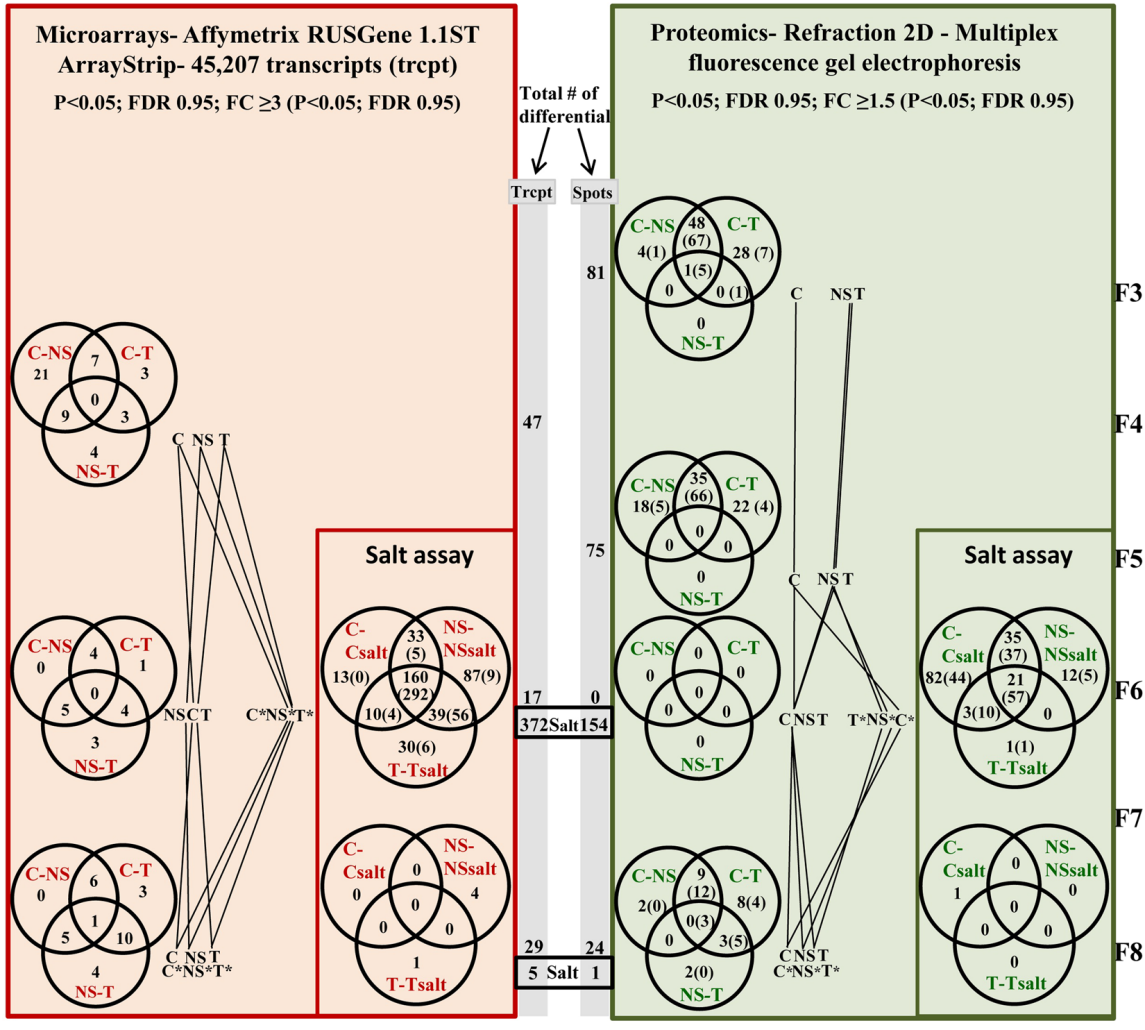
Summary of differentially expressed transcripts/differentially abundant spots throughout generations. Venn Diagrams present the number of differentially abundant spots with a FC ≥ 1.5 and the differentially expressed transcripts with a FC ≥ 3 in each situation. Whenever applied, we present between brackets the redistribution of these spots/transcripts neglecting fold change. For simplification on left panels NS_b_ is represented as NS and T_a_ as T. Trcpt- Transcripts.

**Figure 3:**
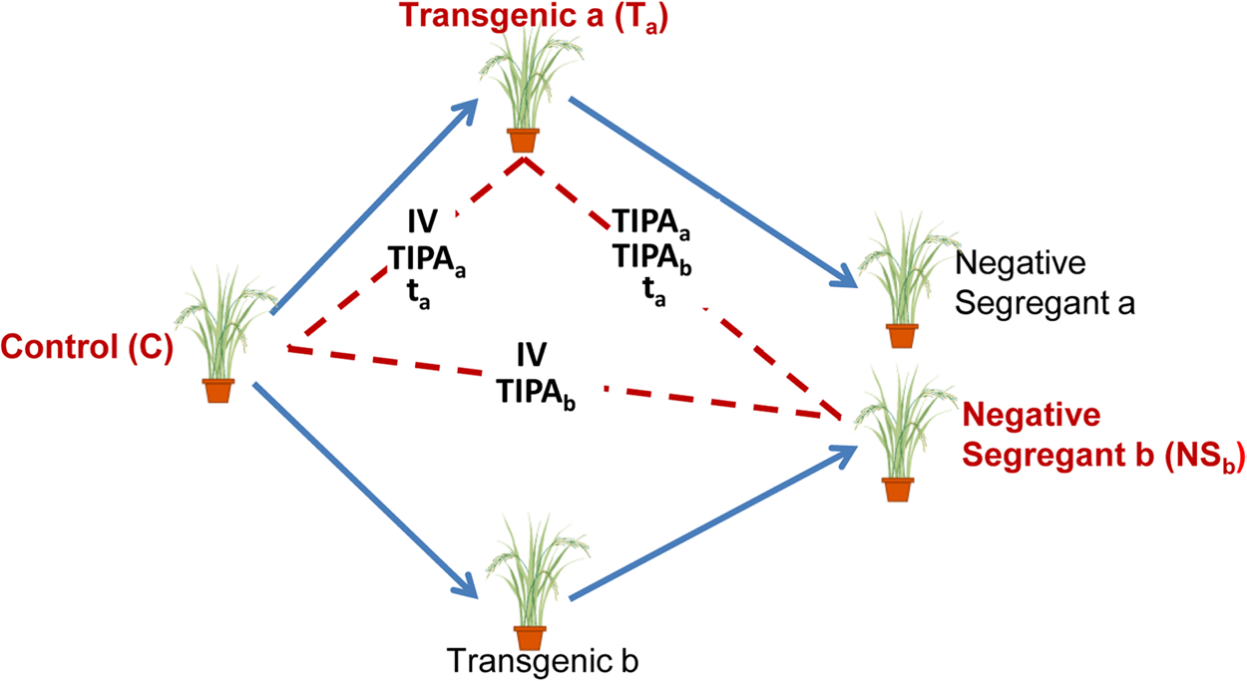
Schematic representation of plant rice lines and the potential factors contributing to their differences. T_a_ and T_b_ were obtained by the same transformation procedure (*Agrobacterium*-mediated). Factors influencing differences between tested plants: **IV**- *In vitro* culture promoted alterations; **TIPA**_**a**_ and **TIPA**_**b**_- Transgene *a* and *b* insertion-promoted alteration, respectively; t_a_- transgene *a* presence-promoted alterations. Lines under test are presented in bold.

In a previous study^2^, we noticed the absence of proteomic differences between the F3 generation of Transgenic (T_a_) and Negative Segregant (NS_b_) lines (only one differentially-abundant spot with fold difference ≥1.5). Moreover, we verified that the differentially-expressed proteome in T_a_ *vs*. Control (C) lines was almost identical to the differentially-expressed proteome in NS_b_ *vs*. C lines (49 spots with fold difference ≥1.5, in both T_a_ and NS_b_ *vs*. C) (Fig. 2, Table 1). Motivated by the proteomic results in the F3 generation (and bearing in mind that *in vitro* culture was the only feature in common between T_a_ and NS_b_ - Fig. 3), we hypothesised that *in vitro* culture was the most relevant factor contributing to the differences found between T_a_ and C^2^. Our hypothesis was further supported by mass spectrometry (MS), which showed that T_a_ and NS_b_ lines had modified metabolic pathways and proteins with altered abundance that were previously described as stress-related^2^. This previous study was performed to address the question of which is the best comparator for the risk assessment of GM plants. Some scientists suggest that negative segregants would be better comparators for genetically modified organisms (GMO) because, opposite to conventional counterparts, they allow discounting putative differences generated by the tissue culture procedures (Fig. 3). On the other hand, some scientists consider that the impact of genetic modification cannot be completely assessed using only negative segregants as comparators, since genetic or epigenetic changes promoted by transgene insertion could still remain after segregation. In this study, to compare with T_a_ and with C, we have selected NS_b_ (a negative segregant originating from a transformation event different from T_a_), so that we could assess how relevant would be the differences promoted by the insertion of the event (instead of the specific transgene). If significant differences had occurred, we should have found significant differences between T_a_ and NS_b_ when analysing the F3 generation (see Fig. 3 for the potential factors contributing to the differences among these lines).

**Table 1:**
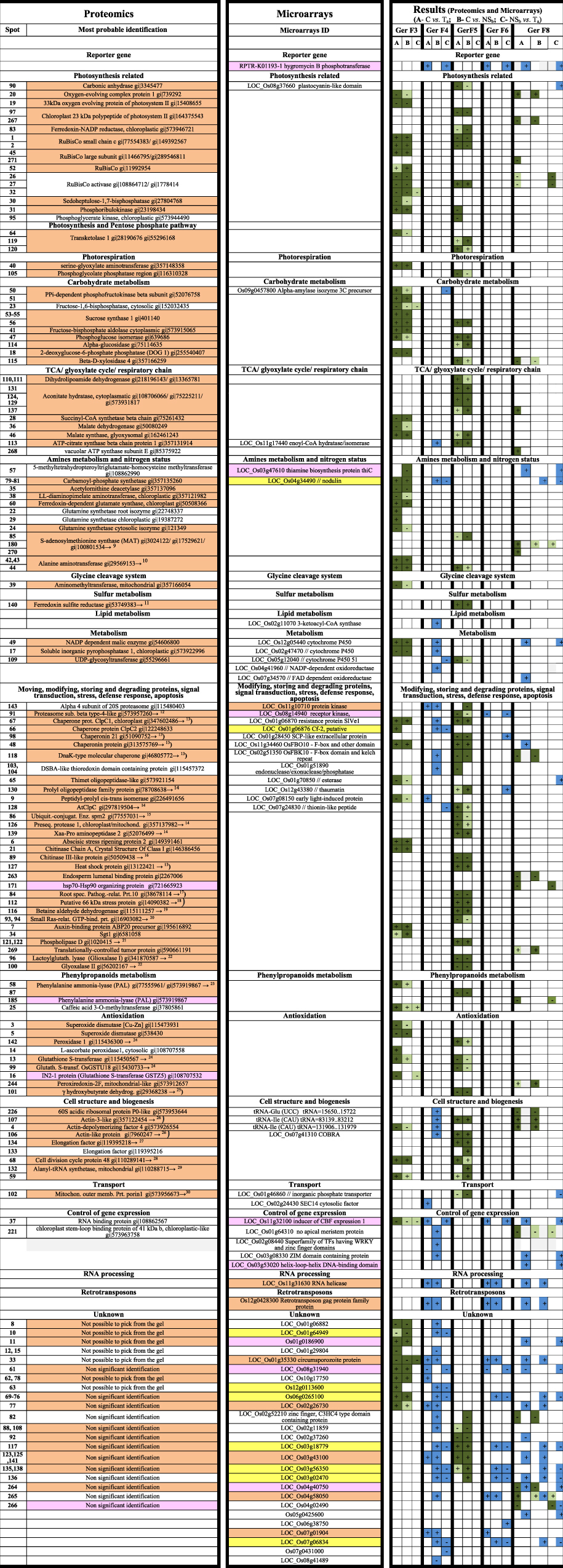
Proteomic and Microarrays results throughout generations (salinity assay not included). Identifications organized by biological process. Proteomics results are presented in green: differentially abundant spots with a FC ≥ 1.5 at least in one of the comparisons (C *vs*.T_a_; C *vs*. NS_b_; NS_b_ *vs*. T_a_); dark green-Fold Change ≥ 1.5; light green- Fold Change < 1.5. Microarrays results are presented in blue: differentially expressed transcripts with a FC ≥ 3 at least in one of the comparisons (C *vs*.T_a_; C *vs*. NS_b_; NS_b_ *vs*. T_a_); all in blue- Fold Change ≥3). Orange shadowed- *in vitro* culture related; Pink shadowed- transgenesis related; Yellow shadowed- negative segregant (NS_b_) specific. Numbers in front of proteins’ identifications regards to references supporting its relation with plant stress. + means up-regulated; − means down-regulated. Note: Protein identifications are independent from transcripts identifications even when on the same table line.

### The number of differentially abundant spots/expressed transcripts changes similarly throughout generations

In this study, we noticed the decrease in differentially-abundant spots throughout generations (Fig. 2). This result is consistent with our previous hypothesis^2^ that proteomic differences found between both T_a_ and NS_b_ *vs*. C, in generation F3, resulted mainly from *in vitro* culture stress. In fact, in generation F6, proteomics could no longer discriminate C, T_a_ and NS_b_ lines (Fig. 2). Surprisingly, on generation F8 the three lines under study could again be discriminated by proteomics although less strongly than on F3 and F5 and with the proximity of NS_b_ and T_a_ in relation to C being less evident (Fig. 2).

To verify if our proteomics data were supported by transcriptomics we analyzed all rice lines using Affymetrix microarrays. We observed that the number of differentially-expressed transcripts between C, T_a_ and NS_b_ throughout generations is extremely low (given the 45,207 transcripts analyzed), namely 47, 17 and 29 on generations F4, F6 and F8, respectively. Reinforcing our proteomics results, F6 was the generation where the three lines under test showed closer microarray profiles, while by generation F8, the three lines slightly diverged as compared to F6 (Fig. 2). This surprising divergence may be due to putative changes occurring sometime along the four years-duration of this experiment, although the environmental conditions were maintained for all plants (temperature, humidity, water supply and periodic greenhouse disinfections), except in generation F6, when the salinity stress was imposed. Still, some uncontrolled factors might have contributed to the observed divergence.

Despite the F8 generation divergence, altogether our data indicate that alterations induced by transgenesis are few and decrease throughout generations.

### Identification of differentially-abundant proteins confirms transgeneration memory of *in vitro* culture stress

To explore the evolution of differentially-abundant spots throughout generations, we evaluated the proteome changes in the three rice lines (T_a_, NS_b_ and C) (Table 1, Table S1). We observed that although the differentially-abundant proteins found between T_a_ and NS_b_ lines *vs*. C clearly changed from F3 to F5, both T_a_ and NS_b_ changed their proteomes in the same direction (Table 1). This seems to confirm that the memory of *in vitro* culture stress on T_a_ and NS_b_ lines is transmitted across generations. In fact, the identified differentially-abundant spots found between T_a_ and NS_b_ *vs*. C, in generation F5, correspond to proteins that could be related to plant stress. Thus, similarly to generation F3, it seems that T_a_ and NS_b_ show decreased photosynthesis efficiency, as carbonic anhydrase (which facilitates CO_2_ supply to RuBisco in C3 plants -spot 90) as well as chloroplast 23 kDa polypeptide of photosystem II (spot 97), ferredoxin-NADP reductase chloroplastic (spot 83) and RuBisCo (spots 1, 2) appear down-regulated. The increased amount of RuBisCo activase (spot 27) may be also related to a decrease in photosynthesis efficiency as already reported by Fukayama *et al*.^7^. However, contrary to generation F3, in F5, there is no clear evidence of photorespiration, or the related enhancement of ammonia re-assimilation pathways. Actually, phosphoglycolate phosphatase (spot 105), which catalyzes the first reaction of the photorespiratory cycle, appears less abundant in T_a_ and NS_b_. Additionally, as in generation F3, the reduction of carbon fixation seems to be compensated by the mobilization of storage substances, such as fatty acids and carbohydrates that may be used to produce energy and eventually other intermediate compounds important for stress sensing and signaling. In fact, some proteins that can be related to carbohydrate catabolism (sucrose synthase- spot 56; phosphoglucose isomerase- spot 47; α-glucosidase- spot 114; β-D-xylosidase- spot 115 and transketolase- spots 119, 120) appeared with higher abundance on T_a_ and NS_b_ lines *vs*. C. The importance of sugars, not only as resources for respiration and metabolic intermediates, but also as signaling molecules, controlling gene expression and development in plants was already discussed by others^8^. The glyoxylate cycle up-regulation (Aconitate hydratase- spots 124, 129, 131, 137; malate synthase- spot 46; ATP-citrate synthase beta chain protein- spot 113) may suggest that plants are using acetate obtained from fatty acids, as source of both carbon and energy. Besides the above reported metabolic adjustments, we also found several other stress-related proteins with altered abundance in T_a_ and NS_b_ lines in generation F5. These proteins and references supporting their stress-relation are listed in Table 1 ^9–30^. As previously stated, proteomics on generation F6 could no longer distinguish the three tested lines. Thus, our analyses confirm that *in vitro* culture stress applied by the time of transgenesis was memorized across generations, and that this “memory” attenuated with time.

### Transcriptome analyses reveal scarce but consistently altered gene-expression throughout generations

To obtain greater insight into the gene regulation throughout generations, we analyzed the transcriptome changes in the three rice lines (T_a_, NS_b_ and C). As mentioned above, we found that the number of differentially-expressed transcripts throughout generations was extremely low, and corresponded mostly to proteins with unknown function (Table 1, Table S2). However, opposite to proteomics data, with the microarrays study, we found transcripts consistently differentially-expressed throughout generations. Moreover, although no longer visible by proteomics, microarrays revealed that *in vitro* culture stress is still memorized on generation F8, as we found three genes likely related to *in vitro* culture stress: circumsporozoite protein precursor (Os01g35330), hrpA-like helicases (Os11g31630) and retrotransposon gag protein family protein (Os12g0428300). These genes showed a fold change (FC) of approximately 4.5, 15, and 7 in NS_b_ and T_a_ *vs*. C_,_ in all three tested generations (F4, F6 and F8). In addition, we found three genes directly related to the transformation event constantly up-regulated in T_a_ throughout generations. These were hygromycin B phosphotransferase (RPTR-K01193-1- FC ≈200) (which was used as a reporter gene in T_a_ transformation), the “inducer of CBF expression” (ICE) 1 (Os11g3210- FC ≈12) (which was the inserted transgene in T_a_), and still another transcript (Os08g31940- FC ≈ 5), which encodes an expressed protein of unknown function. This last mentioned locus is positioned on chromosome (Chr) 8 while the ICE 1 transgene was inserted on Chr 1^2^. Thus, it seems that the up-regulation of Os08g31940 is not related to the transgene insertion site. Moreover, we also found four genes consistently up-regulated in NS_b_, all of them corresponding to proteins of unknown function: Os03g18779- FC ≈10; Os03g56350- FC ≈14; Os07g06834- FC ≈10 and Os06g0265100- FC ≈9.

Hence, the scarce transcriptomic differences found among the three rice lines throughout generations, reinforce the idea that genetic engineering is a highly specific and controlled technology causing very limited pleiotropic alterations.

### Salinity stress highly impacts proteomes and transcriptomes and also affects physiological parameters in the three tested rice lines

**Figure 4:**
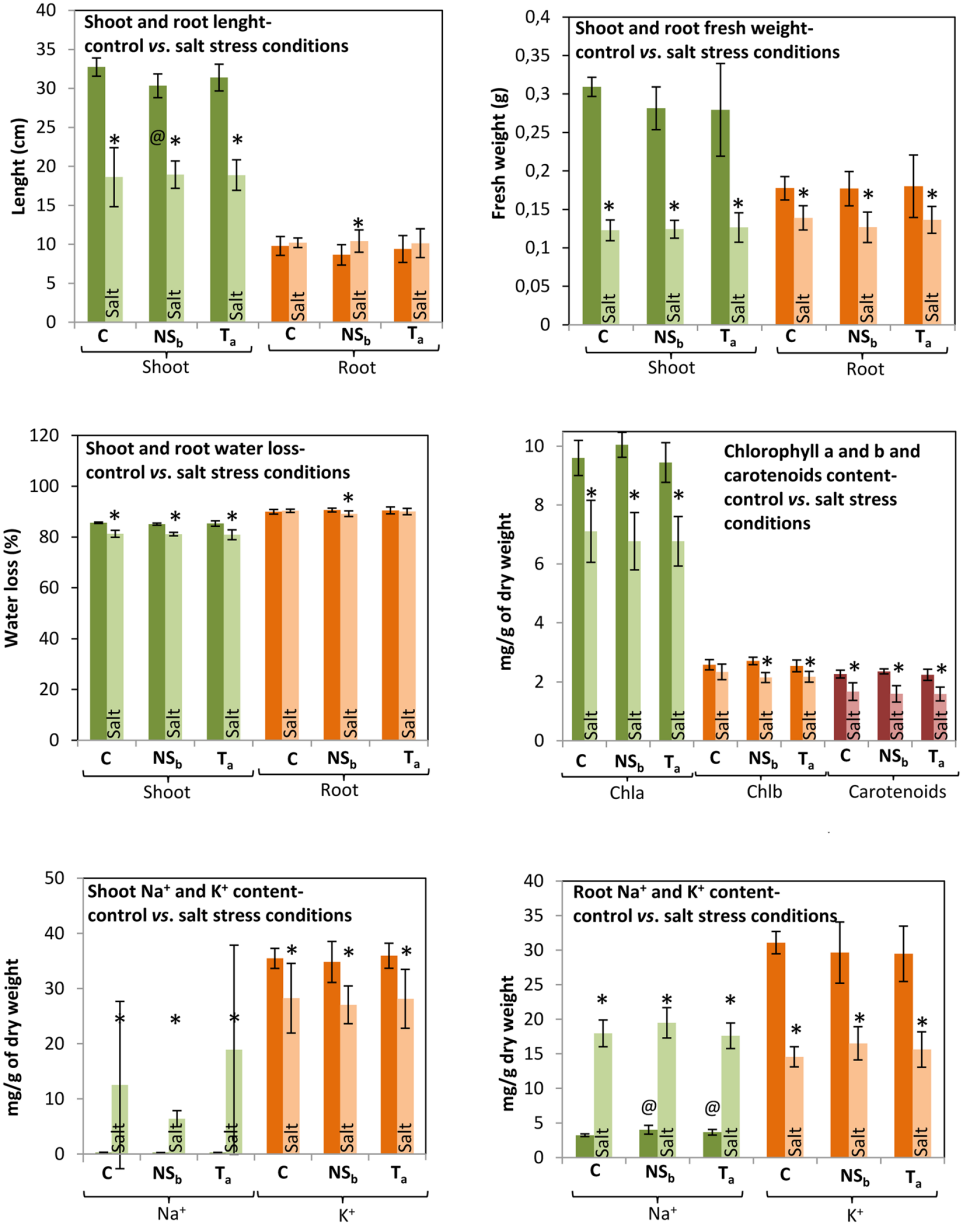
Physiological parameters of F6 plants. For each physiological parameter and plant tissue, statistical significant differences between control and stressed plants, for each line, and among the three rice lines grown in the same environmental conditions, were evaluated. Except for the rice lines highlighted with @ (symbol means statistically different from Control line, p < 0.05) lines grown in the same environmental conditions (C *vs*. T; C *vs*. NS; NS *vs*. T; Csalt *vs*. Tsalt; Csalt *vs*. NSsalt; NSsalt *vs*. Tsalt) present no statistically different physiological parameters. Asterisks (*) represent statistically significant differences (p < 0.05) between control *vs*. stressed plants (C *vs*. Csalt; NS *vs*. NSsalt and T *vs*. Tsalt). Error bars represent standard deviation.

To evaluate the dimension of the transcriptomic and proteomic alterations observed throughout generations with the ones that may be induced due to environmental stress, we performed a salinity assay in F6 generation and analyzed the proteome and transcriptome of T_a_, C and NS_b_. As expected, we confirmed that salinity stress strongly influenced rice proteomes/transcriptomes. We found 154 spots differentially-abundant with a FC > 1.5, and 372 transcripts differentially-expressed with a FC > 3 at least in one of the three comparisons (C *vs*. Csalt; NS_b_ *vs*. NS_b_salt and T_a_ *vs*. T_a_salt). Fifty seven out of the 154 differentially-abundant spots (37%) and 292 out of the 372 differentially-expressed transcripts (78%) were changed in the three lines as a consequence of the salt stress (Fig. 2). Also, with the exception of root length, root water loss, and Chlorophyll b content, all tested physiological parameters (shoot length, root and shoot biomass, shoot water loss and carotenoids, chlorophyll a, Na^+^ and K^+^ contents) were statistically different in all tested rice lines in control *vs*. stress conditions (C *vs*. Csalt; NS *vs*. NSsalt and T *vs*. Tsalt – Fig. 4).

**Figure 5:**
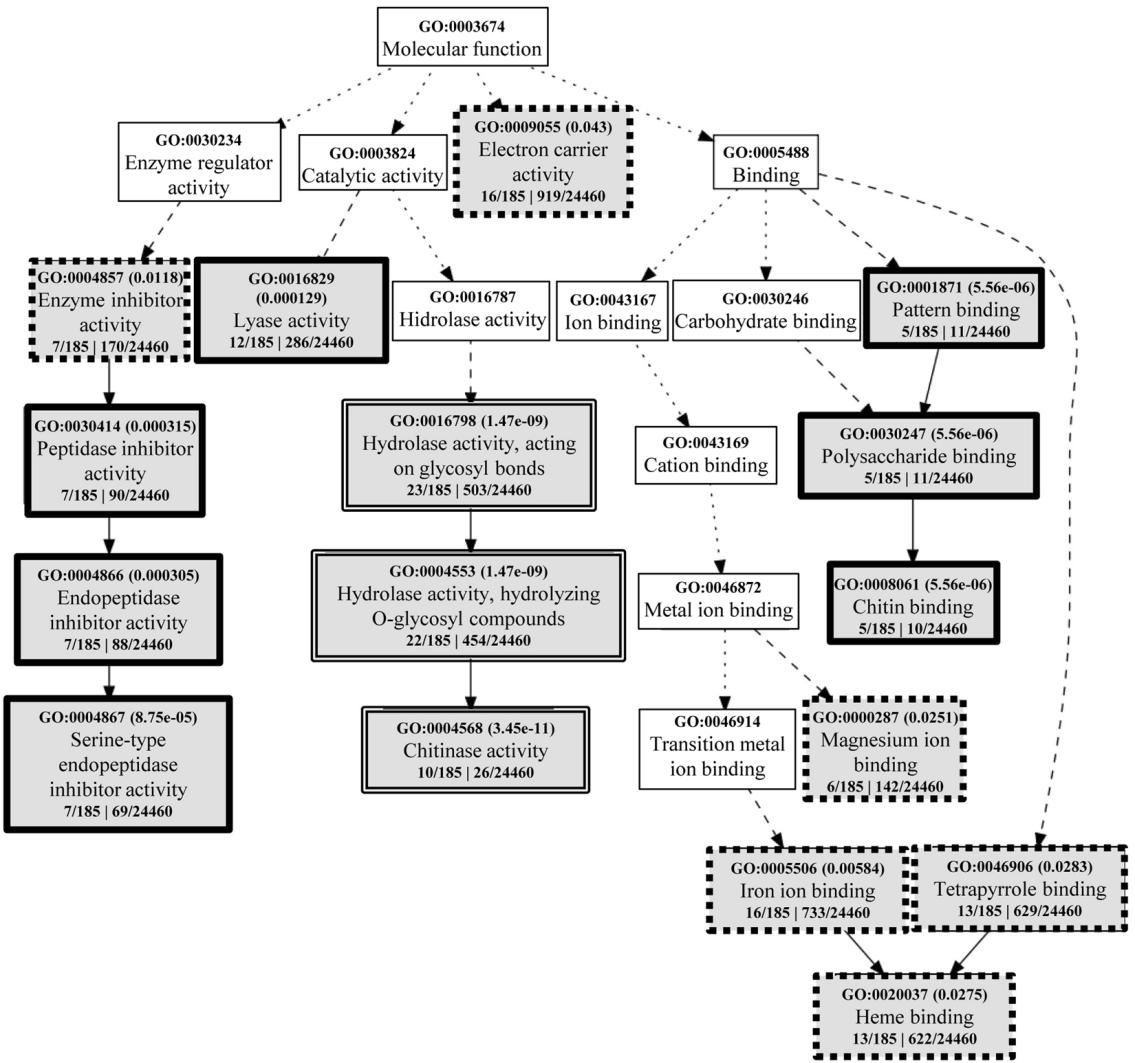
Molecular Function Gene Ontology (GO) analysis of rice salt-stress induced genes using agriGO. Differentially expressed transcripts in all three tested situations (C *vs*. Csalt, NS *vs*. NS salt and T *vs*. T salt) and with FC > 3 at least in one of the comparisons were considered (292 transcripts). One hundred and eighty five transcripts could be annotated in agriGO. Each box shows the GO term number and the p-value in parenthesis. The first pair of numerals represents the ratio between the number of genes in the input list associated with that GO term and the total number of agriGO annotated genes in the input list (185). The second pair of numerals represents the number of genes associated with the particular GO term in the MSU Rice Genome annotation Project Database Resource and the total number of rice genes with GO annotations in the same rice database. The box line indicates levels of statistical significance with ■■■■ = 0.05; ▬▬ = e-05 and ══ = e-09.

To improve the analysis of the most salt-affected biological processes, we used agriGO singular enrichment analysis (SEA)^31^. A total of 292 transcripts, which appeared differentially-regulated in all three comparisons, were analyzed with agriGO (Fig. 5, Table S2). A total of 185 out of the 292 differentially-expressed transcripts retrieved agriGO annotations, and the most enriched molecular functions-related GO terms were: GO:0016798- hydrolase activity, acting on glycosyl bonds, and, as a part of this, GO:0004568- chitinase activity (Fig. 5). Glycoside hydrolases, which are involved in the degradation and reorganization of cell wall polysaccharides, as well as chitinases, have been largely described as related to stress response in plants^16, 32^. Other GO terms with a false discovery rate (FDR) <0.05 were GO:0004867- serine-type endopeptidase inhibitor activity, GO:0016829- lyase activity and GO:0008061- chitin binding, which appeared enriched by salinity stress. The chitin binding GO term enrichment was, obviously, related to the already mentioned chitinase activity enriched GO term. Serine-type endopeptidase inhibitor activity enrichment was also not surprising because peptidase inhibitor activity in plants is largely related to the response to plant protection against insects, pathogens and environmental stresses^33^. Lyase activity GO term enrichment was essentially based on the differential expression of 12 transcripts, six of them related to terpene biosynthesis (five corresponding to terpene synthases and one to ent-kaurene synthase). It is widely known that terpenoids play important roles in plant interactions with the environment and in concomitant stress responses^34^. Finally, other GO terms enriched by salt were the electron carrier activity (GO:0009055), iron ion binding (GO:0005506), heme binding (GO:0020037), tetrapyrrole binding (GO:0046906) and magnesium ion binding (GO:0000287). The enrichment of iron ion binding (16 identifications), tetrapyrrole binding (13 identifications), heme binding (13 identifications) and electron carrier activity (16 identifications) molecular functions, was essentially related to the differential expression of seven transcripts corresponding to cytochrome P450 and five transcripts corresponding to peroxidase. It is well established that, to some degree, virtually all biotic and abiotic stresses induce, or involve, oxidative stress, and that peroxidases are involved in the control of H_2_O_2_ levels^24, 35^. Cytochrome P450 has been described as involved in the crosstalk between responses to abiotic and biotic stresses, and its expression may be regulated by ROS-related signalling pathways^36^. Magnesium ion binding molecular function enrichment seems to be related to terpene biosynthesis because the six differentially-regulated transcripts involved in this pathway have magnesium ion binding function.

**Figure 6:**
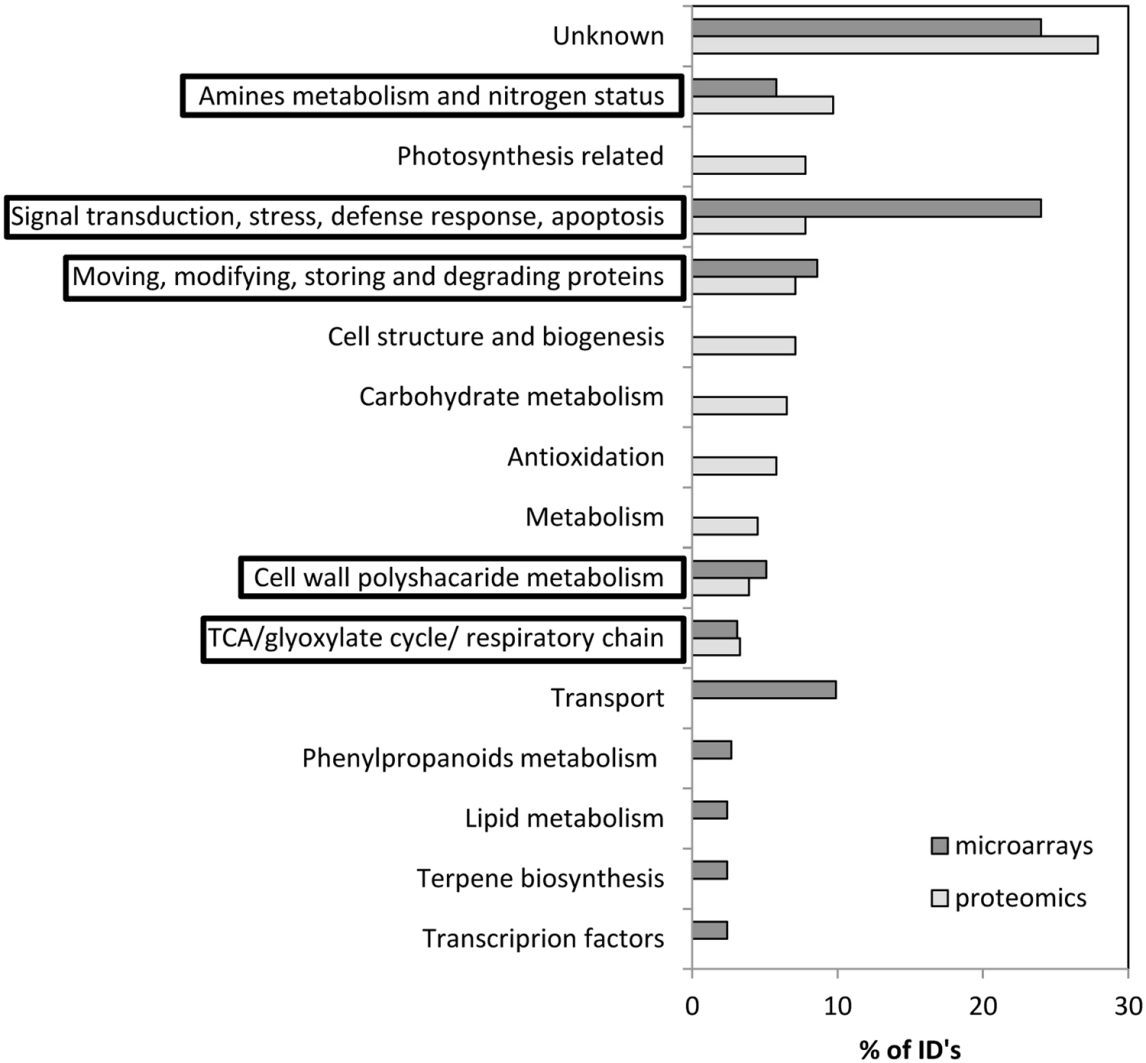
The ten most representative salt responsive molecular functions categories identified by Proteomics and Microarrays. Percentage of identifications in each category in relation to the total number of salt responsive identifications. Molecular function categories in black boxes correspond to the ones coincident in Proteomics and Microarrays data.

The application of agriGO analysis tool to our proteomics data could only retrieve annotations for 11 out of the 154 salt-responsive proteins. Thus, we sought to establish a correlation between the microarrays and proteomics data of the F6 generation salinity assay data (Table S3). The correspondence between the observed salt responsive differentially-abundant spots and differentially-expressed transcripts was scarce. The only identifications with correspondence and identical response (up-regulation) were chitinase, heat shock proteins, thaumatin and actin-depolymerizing factor. Carbonic anhydrase, fructose-bisphosphate aldolase, glycosyl hydrolase and peroxidase were also identified as differentially-abundant proteins and differentially-expressed transcripts, but with contrasting responses (up-regulation in one case and down-regulation in the other). Figure 6 presents the ten most representative salt-responsive molecular functions categories identified by proteomics and microarrays as the percentage of identifications in each category in relation to the total number of salt responsive identifications. This analysis confirms that proteomics and microarrays results of salinity assay are different although five out of the ten categories overlaps. These include “Signal transduction, stress response and apoptosis”, “Moving, modifying, storing and degrading proteins”, “Amine metabolism and nitrogen status”, “Cell wall polysaccharide metabolism” and “TCA/glyoxylate cycle/respiratory chain” molecular function categories. The high abundance of differentially-abundant/expressed proteins/transcripts related to the modification and degradation of proteins could explain the differences between proteomics and microarrays data, as many proteins may be regulated at post-translational level^37^.

Because of the public concern about the putative increase of allergenicity associated to genetically modified plants, we sought to predict the allergenicity of salt-responsive proteins. In this way, we wanted to check if environmental stress could be related to increased levels of potential allergenic proteins. Interestingly, a total of 56 out of the 112 (50%) identified salt-responsive proteins were potential allergens and 112 out of the 292 (38%) differentially-expressed transcripts are translated into proteins that are putative allergens, using Algpred software^38^ (Table S3). Although this prediction does not prove allergenicity, the results certainly demonstrate that environmental stimuli *per se* may strongly impact the allergenicity of a given plant food.

Notably, both proteomics and microarrays analysis of stressed *vs*. non-stressed plants on generation F8 (2 generations after salt stress imposition) show that salt stress has been almost completely “forgotten”, as the salt-induced alterations almost disappeared. In this generation, we found only one differentially-abundant protein (hsp70-Hsp90 organizing protein- gi|721665923) and five differentially-expressed genes (four unknown- Os04g40750, Os04g22940, Os10g21190, Os04g05659 and one alpha-amylase precursor- Os02g52700) in stressed *vs*. non-stressed rice lines (Fig. 2). Thus, we may conclude that salinity stress affected in the same way all three rice lines, leading to significant changes not only in several physiological parameters, but also in the expression/abundance of the typical stress-responsive genes/proteins.

We have demonstrated that *in vitro* culture stress seems to be the key factor responsible for the differences between transgenic *vs*. non-transgenic control plants. Our proteomics data corroborates that T_a_ and NS_b_ lines have their expression similarly modified throughout generations, both becoming biochemically closer to control plants. This fact suggests that differences promoted during genetic modification seem to be mainly short-term changes that become attenuated throughout generations. In addition, as expected, our results clearly show that salinity stress strongly affects plants’ response with consequences at physiological, transcriptional and proteomic levels. In this study, the impact of genetic engineering was assessed two generations after plant transformation, while the impact of salinity stress was evaluated immediately after stress imposition. However, the current regulatory measures impose that transgenic plants are submitted to exhaustive tests throughout several generations before coming to the market. The evaluation of “the genetic stability of the inserted/modified sequence” and “the phenotypic stability of the GM plant” are examples of tests that require several generations to accomplish; in these particular cases, applicants need to provide data from usually five generations or vegetative cycles. Thus, genetically engineered plants that enter the market have already several generations after the transgenesis event. On the contrary all crops (transgenic or not) are unavoidably exposed to different biotic/abiotic stresses (eg: temperature variations, drought, UV, pathogens, phytochemicals, post-harvest stress), and consequently all consumers eat plants affected by environmental stresses. Because stress conditions may strongly influence the production of proteins eventually toxic/allergenic^3–6^, it is likely that plant foods toxicity and/or allergenicity vary depending on growth/processing conditions.

If, in fact, environment *per se* can originate far more safety issues than genetic engineering, we believe it is pertinent to question what is really relevant and what is clearly excessive when designing risk assessment for Genetically Modified Organisms.

## Methods

### Plant material

Three lines of *Oryza sativa* L. ssp. *japonica* cv. Nipponbare were used, as previously described^2^: a seed-derived control line (C); a GM line obtained by *Agrobacterium*-mediated transformation (T_a_), and a non-GM negative segregant control line (NS_b_, progeny of Transgenic b) (Fig. 3). Ta carried one copy of OsICE1 transcription factor, fused with a TAP- tag construction, driven by the maize ubiquitin promoter. NSb was a F1 descendent of a transgenic line (Tb) which also contains OsICE1 transcription factor driven by maize ubiquitin promoter. However, TAP-tag was not present in the construct introduced in Tb. All lines (C, T_a_, NS_b_) were grown in the same conditions throughout generations (soil:peat:vermiculite (2:2:1); 28 °C/24 °C (light/night); 12 h photoperiod; 70% relative humidity).

### Seed treatment and seedling growth for Proteomics and Microarrays

Rice seeds were treated at 50 °C in 0.1% Carbendazim (fungicide) for 30 min, and surface disinfected using standard procedures. Seeds were germinated in darkness, for two days at 28 °C. Seedlings were grown for 13 days in Yoshida medium^39^, at the above conditions (temperature, photoperiod and humidity). Fifteen days-old seedlings were frozen in liquid nitrogen and kept at −80 °C until RNA/protein extractions.

Transgene presence was PCR-confirmed for T_a_ seedlings as previously described^2^.

### Salinity stress imposition and physiological evaluation on F6 generation plants

Salinity stress (12 dS/m final concentration) was imposed on half of the seedlings (C, T_a_ and NS_b_) in F6 generation according to IRRI protocol^40^. Fifteen days-old seedlings were collected after 12 days of salinity stress imposition. From F6 generation onwards, we worked with six rice lines (C, Csalt, T_a_, T_a_salt, NS_b,_ NS_b_salt).

Twenty-four seedlings of each line (C, Csalt, T_a_, T_a_salt, NS_b_ and NS_b_salt) were used for physiological evaluation, namely nine seedlings for root/shoot length, biomass and water loss percentage; six for carotenoids and chlorophyll determination; for Na and K content, the root and the third leaf of nine seedlings were used. Chlorophyll (Chl) *a*, Chl*b* and carotenoids (Car) were determined on the third leaf using UV-Vis spectroscopy after whole pigment extraction with 80% acetone (Chl*a* = 12.25 × A_663.2_ − 2.79 × A_646.8_; Chl*b* = 21.5 × A_646.8_ − 5.1 × A_663.2_; Car = ((1000 × Abs_470_) − (1.82 × Chla) − (85.02 × Chlb))/198)^41^. Na and K contents were determined using atomic emission spectroscopy after extraction with acetic acid 0.1N.

The comparison of physiological parameters among control vs. stressed plants (C vs. Csalt; NSb vs. NSbsalt and Ta vs. Tasalt) was achieved by two-tail T-test (p < 0.05). For the comparison of the physiological parameters among the three rice lines grown in the same environmental conditions (C vs. Ta; C vs. NSb; NSb vs. Ta; Csalt vs. Tasalt; Csalt vs. NSbsalt; NSbsalt vs. Tasalt) one-way ANOVA and, when significant differences were found (p < 0.05), subsequent two-tail T test (with a corrected p value of 0.05/3 = 0.0167) was performed.

### Protein Extraction and RuBisCO depletion

Protein was extracted by standard procedures from three pools of six (generation F3) or 10 (generations F5, F6 and F8) whole-seedlings at 4^th^-leaf stage, and RuBisCO depletion was performed as previously described^2^. The resulting RuBisCO depleted pellets were dissolved in Lysis Buffer (30 mM Tris-HCl (pH 8.5), 7M Urea, 2M Thiourea, 4% CHAPS). The protein was measured according to Ramagli^42^ using albumin from chicken egg white (Sigma) as a standard.

### Refraction 2D- multiplex fluorescence gel electrophoresis technology

Fifty micrograms of protein, of each of the three biological replicates, and each of the three (C, T_a_, NS_b_), or six (C, T_a_, NS_b_, Csalt, T_a_salt, NS_b_salt), rice lines was labelled according to the manufacturer’s instructions (NH DyeAgnostics) with G-Dye300 and G-Dye200 dyes. An internal standard (containing the same amount of all samples) labelled with G-Dye100, was used to normalize spot volumes and support gel alignment procedures.

Proteins were separated by 2D PAGE using 13-cm-long, 3–11 NL, IPG strips (Amersham Biosciences). Strips passive rehydration, focusing and equilibration were performed as previously described^2^.

SDS-PAGE was performed on 12.5% T, 1.4% C gels in a Hoefer SE 600 system (Amersham Biosciences) and run at 15 °C with a 15 mA/gel constant current for 15 min, and then at 30 mA/gel.

### Gel imaging and statistical analyses

Gels’ images were acquired following manufacturer’s instructions (DyeAgnostics), using a Fuji FLA-5100. Each scan was optimized to maximum dynamic range without reaching image saturation. Images quality control, visual validation of differentially-abundant spots, as well as subsequent statistical analysis, was performed using Progenesis Samespots (v4.5) software. Identification of differentially abundant proteins was performed by one way ANOVA analyses of the spots-normalized volume. Spots with fold differences ≥1.5, Anova p-values < 0.05, and a false discovery rate (FDR) with a 0.05 threshold (q-value < 0.05), were selected.

Figure S1 shows a typical gel, and identifies the 271 spots with differential abundance, and FC ≥ 1.5 in at least one of the tested situations.

### MS analysis and database search

Spots of interest were automatically digested and spotted with an Evo 2 workstation (Tecan).

Peptide mass determinations was performed using the 5800 Proteomics Analyzer (ABsciex) in reflectron mode for both peptide mass fingerprint and MS/MS. Proteins were identified by searching the MS and MS/MS data against NCBI database, (downloaded on September 26, 2011) in the viridiplantae taxonomy (898660 sequences). Database search was performed using an in house MASCOT 2.3 server (www.matrixscience.com) as previously described^43^. Proteins were identified with a minimum of two MS/MS spectra matching the databank sequence and a total ion score above 60. All protein identifications were manually validated.

### Microarrays analysis

RNA from three pools of ten whole-seedlings was isolated using RNeasy Plant Mini Kit (Qiagen) following the manufacturer’s instructions. Total RNA was kept at −80 °C until labeling and hybridization to the Affymetrix RUSGene 1.1ST ArrayStrip at the Affymetrix core facility (Instituto Gulbenkian de Ciência, Oeiras, Portugal). This array contains 816815 probes to query 45207 transcripts. Gene level normalization and signal summarization was performed on Affymetrix Expression Console Software. Chipster software was used for gene filter by standard deviation (Bayes t-test, p value < 0.05) and for Benjamini and Hochberg’s false discovery rate test (0.05 threshold) of filtered data.

AgriGO database^31^ was used with default parameters for functional annotation and enrichment analysis through gene ontology of salt stress-responsive transcripts differentially expressed in the three comparisons (C *vs*. Csalt, NS_b_ *vs*. NS_b_salt and T_a_ *vs*. T_a_salt), and presenting an FC > 3 at least in one of the comparisons.

Because many proteins of the plant defence system are also allergens, we used the AlgPred hybrid approach^38^ to predict potential allergenicity of salt-responsive proteins with differential abundance, or encoded by differentially-expressed transcripts.

### Data availability

Microarrays data was deposited on the Gene Expression Omnibus (GEO)- GSE85113: Expression data from three rice lines (1-control, 1-transgenic and 1-negative segregant) throughout generations and under salt.

## Electronic supplementary material

Supplementary materials

Table S1

Table S2

## References

[1] EFSA P on Genetically Modified Organisms. Scientific opinion on Guidance for risk assessment of food and feed from genetically modified plants. *EFSA Journal* **9**, 2150 [pp. 37] (2011**)**.

[2] Fonseca C (2015). *In vitro* culture may be the major contributing factor for transgenic *versus* nontransgenic proteomic plant differences. Proteomics.

[3] Ahlholm JU, Helander ML, Savolainen J (1998). Genetic and environmental factors affecting the allergenicity of birch (Betula pubescens ssp. czerepanovii [Orl.] Hämet-ahti) pollen. Clinical and Experimental Allergy.

[4] Hänninen A (1999). -riitta, Mikkola, J. H., Kalkkinen, N., Ylitalo, L., Reunala, T. & Palosuo, T. Increased allergen production in turnip (*Brassica rapa*) by treatments activating defense mechanisms. Journal of Allergy and Clinical Immunology.

[5] Pühringer H (2000). The promoter of an apple Ypr 10 gene, encoding the major allergen Mal d 1, is stress- and pathogen-inducible. Plant Science.

[6] Armentia A, Callejo A, Díaz-Perales A, Martín-Gil FJ, Salcedo G (2003). Enhancement of tomato allergenicity after treatment with plant hormones. Allergologia et Immunopathologia.

[7] Fukayama H (2012). Overexpression of Rubisco Activase Decreases the Photosynthetic CO_2_ Assimilation Rate by Reducing Rubisco Content in Rice Leaves. Plant Cell Physiol.

[8] Rosa M (2009). Soluble sugars — Metabolism, sensing and abiotic stress. Plant Signaling & Behavior.

[9] Breusegem FV, Dekeyser R, Gielen J, Montagu MV, Caplan A (1994). Characterization of a S-Adenosylmethionine Synthetase Gene in Rice. Plant Physiol.

[10] Kendziorek M, Paszkowski A, Zagdanska B (2012). Differential regulation of alanine aminotransferase homologues by abiotic stresses in wheat (Triticum aestivum L.) seedlings. Plant Cell Reports.

[11] Yarmolinsky D (2014). Impairment in Sulfite Reductase Leads to Early Leaf Senescence in Tomato Plants. Plant physiology.

[12] Kurepa J, Wang S, Li Y, Smalle J (2009). Proteasome regulation, plant growth and stress tolerance. Plant Signaling & Behavior.

[13] Wang W, Vinocur B, Shoseyov O, Altman A (2004). Role of plant heat-shock proteins and molecular chaperones in the abiotic stress response. Trends in Plant Science.

[14] Xia Y (2004). Proteases in pathogenesis and plant defence. Cellular Microbiology.

[15] Lyzenga WJ, Stone SL (2012). Abiotic stress tolerance mediated by protein ubiquitination. Journal of Experimental Botany.

[16] Takenaka Y, Nakano S, Tamoi M, Sakuda S, Fukamizo T (2009). Chitinase Gene Expression in Response to Environmental Stresses in *Arabidopsis thaliana*: Chitinase Inhibitor Allosamidin Enhances Stress Tolerance. Biosci Biotechnol Biochem.

[17] Hashimoto M (2004). A Novel Rice PR10 Protein, RSOsPR10, Specifically Induced in Roots by Biotic and Abiotic Stresses, Possibly via the Jasmonic Acid Signaling Pathway. Plant Cell Physiol.

[18] Mishra AK, Puranik S, Prasad M (2012). Structure and regulatory networks of WD40 protein in plants. Journal of Plant Biochemistry and Biotechnology.

[19] Fitzgerald TL, Waters DLE, Henry RJ (2009). Betaine aldehyde dehydrogenase in plants. Plant biology.

[20] Ruanjaichon V (2004). Small GTP-Binding Protein Gene Is Associated with QTL for Submergence Tolerance in Rice 1. Russian Journal of Plant Physiology.

[21] Bargmann BO, Munnik T (2006). The role of phospholipase D in plant stress responses. Current Opinion in Plant Biology.

[22] Kaur C, Ghosh A, Pareek A, Sopory SK, Singla-Pareek SL (2014). Glyoxalases and stress tolerance in plants. Biochem Soc Trans.

[23] Dixon RA, Paiva NL (1995). Stress-lnduced Phenylpropanoid Metabolism. The Plant Cell.

[24] Gill SS, Tuteja N (2010). Reactive oxygen species and antioxidant machinery in abiotic stress tolerance in crop plants. Plant Physiology and Biochemistry.

[25] Breitkreuz KE (2003). Novel gamma-Hydroxybutyrate Dehydrogenase. The Journal of Biological Chemistry.

[26] Henty-Ridilla JL (2013). The Plant Actin Cytoskeleton Responds to Signals from Microbe-Associated Molecular Patterns. PLoS Pathogens.

[27] Bhadula SK (2001). Heat-stress induced synthesis of chloroplast protein synthesis elongation factor (EF-Tu) in a heat-tolerant maize line. Planta.

[28] Marshall RS (2008). The Role of CDC48 in the Retro-translocation of Non-ubiquitinated Toxin Substrates in Plant Cells. The Journal of Biological Chemistry.

[29] Szymanski M, Deniziak M, Barciszewski J (2000). The new aspects of aminoacyl-tRNA synthetases. Acta Biochimica Polonica.

[30] Tateda C, Watanabe K, Kusano T, Takahashi Y (2011). Molecular and genetic characterization of the gene family encoding the voltage-dependent anion channel in Arabidopsis. Journal of Experimental Botany.

[31] Du Z, Zhou X, Ling Y, Zhang Z, Su Z (2010). agriGO: a GO analysis toolkit for the agricultural community. Nucleic Acids Research.

[32] Minic Z, Jouanin L (2006). Plant glycoside hydrolases involved in cell wall polysaccharide degradation. Plant Physiology and Biochemistry.

[33] Habib H, Fazili KM (2007). Plant protease inhibitors: a defense strategy in plants. Biotechnology and Molecular Biology Review.

[34] Pateraki I, Kanellis AK (2010). Stress and developmental responses of terpenoid biosynthetic genes in Cistus creticus subsp. creticus. Plant Cell Reports.

[35] Cheeseman JM (2007). Hydrogen Peroxide and Plant Stress: A Challenging Relationship. Plant Stress.

[36] Narusaka Y (2004). Crosstalk in the responses to abiotic and biotic stresses in Arabidopsis: Analysis of gene expression in cytochrome P450 gene superfamily by cDNA microarray. Plant Molecular Biology.

[37] Guerra D (2015). Post-transcriptional and post-translational regulations of drought and heat response in plants: a spider’s web of mechanisms. Frontiers in Plant Science.

[38] Saha S, Raghava GPS (2006). AlgPred: prediction of allergenic proteins and mapping of IgE epitopes. Nucleic Acids Research.

[39] Yoshida, S., Forno, D. A., Cock, J. H. & Gomez, K. A. No Title. *International Rice Research Institute, Manila, Philippines* (1976).

[40] Gregorio, G. B., Senadhira, D. & Mendoza, R. D. Screening Rice for Salinity Tolerance. *IRRI discussion paper series* NO. 22 (1997).

[41] Inskeep WP, Bloom PR (1985). Extinction Coefficients of Chlorophyll a and b in N, N-Dimethylformamide and 80% Acetone. Plant physiology.

[42] Ramagli LS (1998). Quantifying protein in 2D-PAGE solubilization buffers. Methods in Molecular Biology.

[43] Szopinska A (2016). Stuck at work? Quantitative proteomics of environmental wine yeast strains reveals the natural mechanism of overcoming stuck fermentation. Proteomics.

