# Supplementary material for: Environmental stress is the major cause of transcriptomic and proteomic changes in GM and non-GM plants
Source: Sci Rep. 2017 Sep 6;7:10624. doi: 10.1038/s41598-017-09646-8 (PMC5587699; doi:10.1038/s41598-017-09646-8)
Supplement: Supplementary file 1 — Supplementary materials [file 41598_2017_9646_MOESM1_ESM.pdf]

Title: Environmental stress is the major cause of transcriptomic and proteomic changes in GM and non-GM plants

Author names: Rita Batista, Cátia Fonseca, Sébastien Planchon, Sónia Negrão, Jenny Renaut, M. Margarida Oliveira

Legends for Supporting information

Figure S1- Representative 2D- multiplex fluorescence gel with the 271 differentially-abundant spots and 2D gel sections showing examples of different spot patterns between tested samples.

Table S1- Proteomic results.

Table S2- Microarrays results.

Table S3- Proteomics and Microarrays relation (salinity assay).

Figure S1- Representative 2D- multiplex fluorescence gel with the 271 differentially-abundant spots and 2D gel sections showing examples of different spot patterns between tested samples.

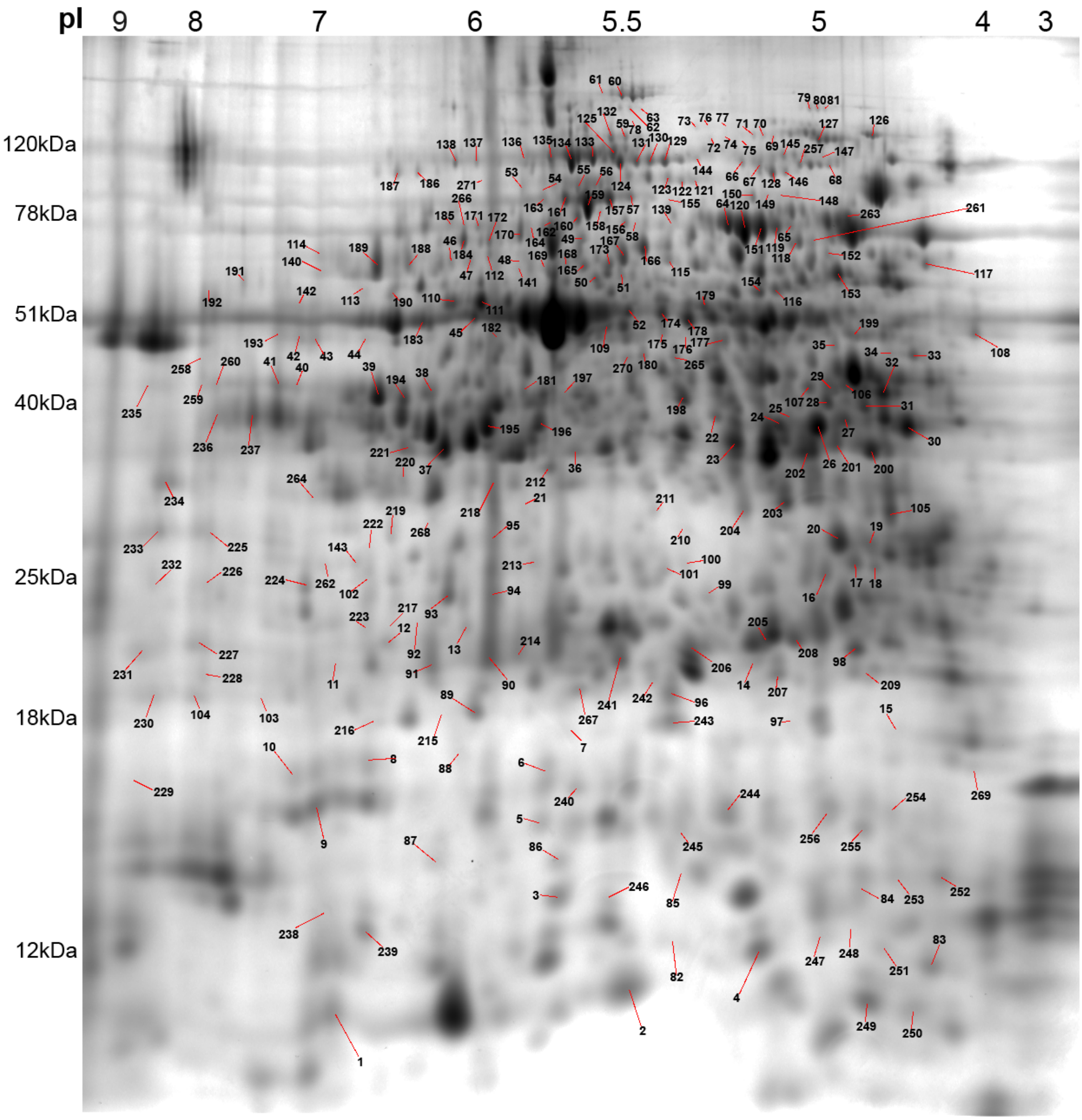

Ger F3

Spot 13- Ger F3

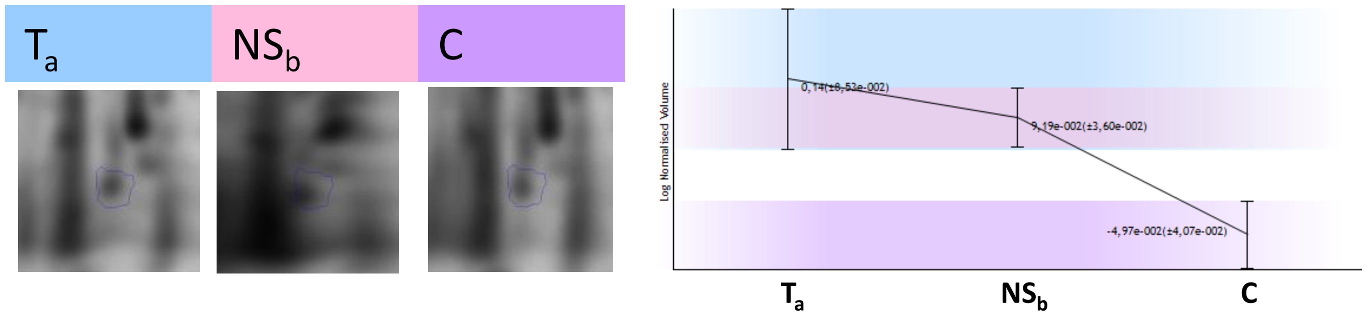

Spot 26- Ger F3

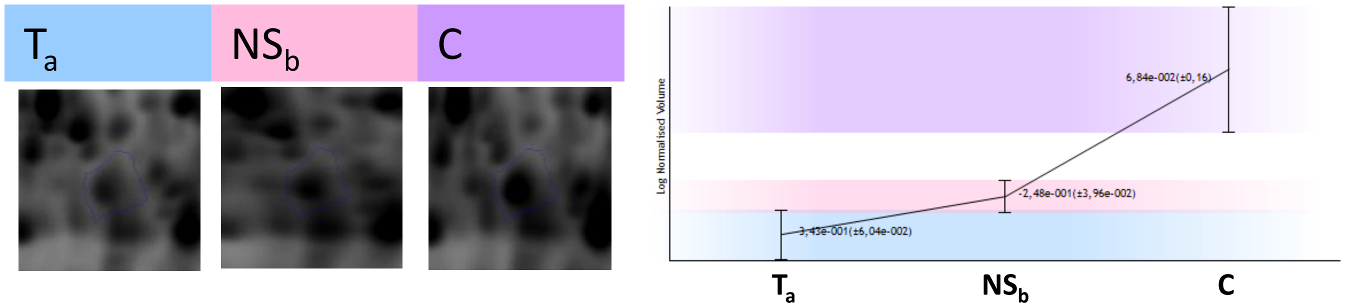

Spot 27- Ger F3

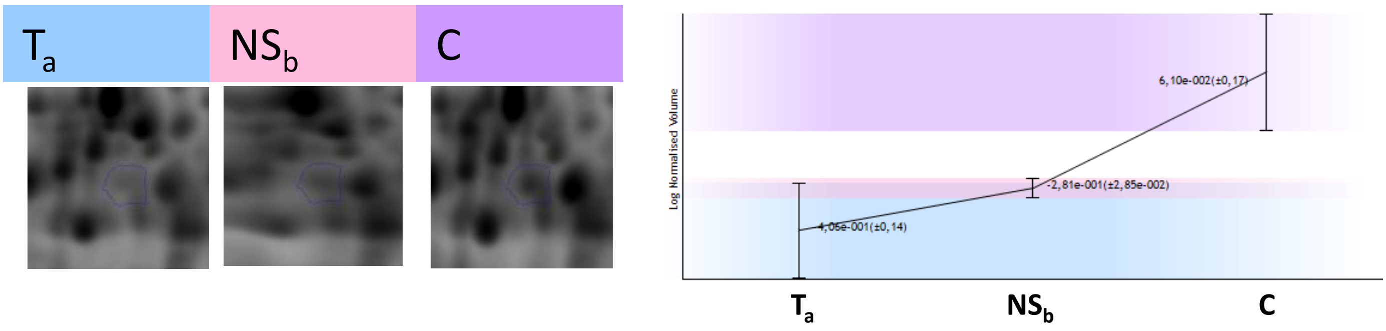

Spot 64- Ger F3

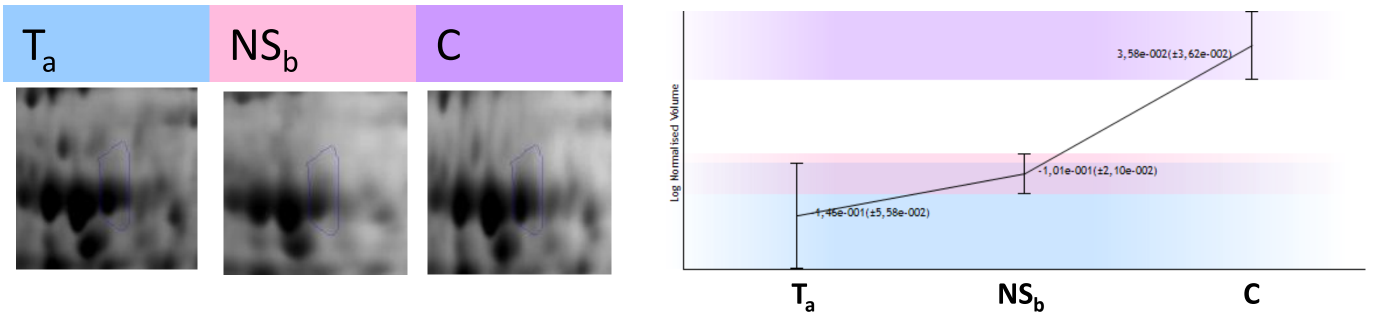

Spot 69- Ger F3

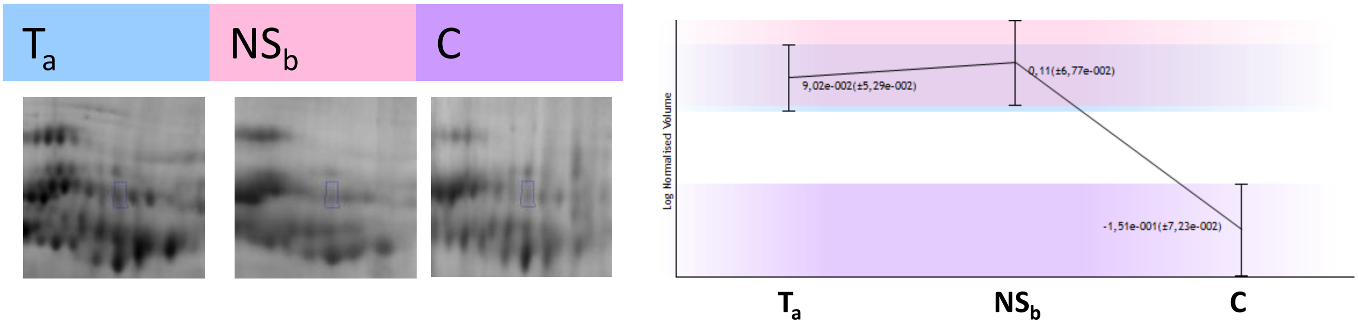

Ger F5

Spot 97- Ger F5

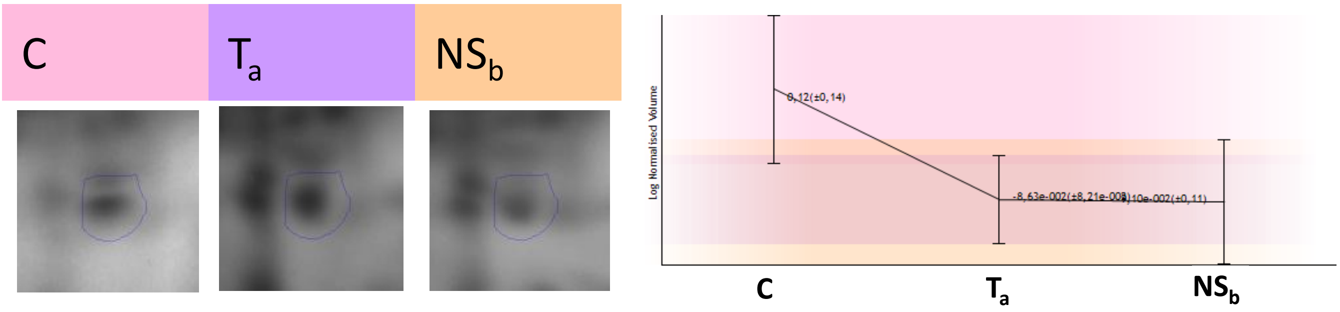

Spot 99- Ger F5

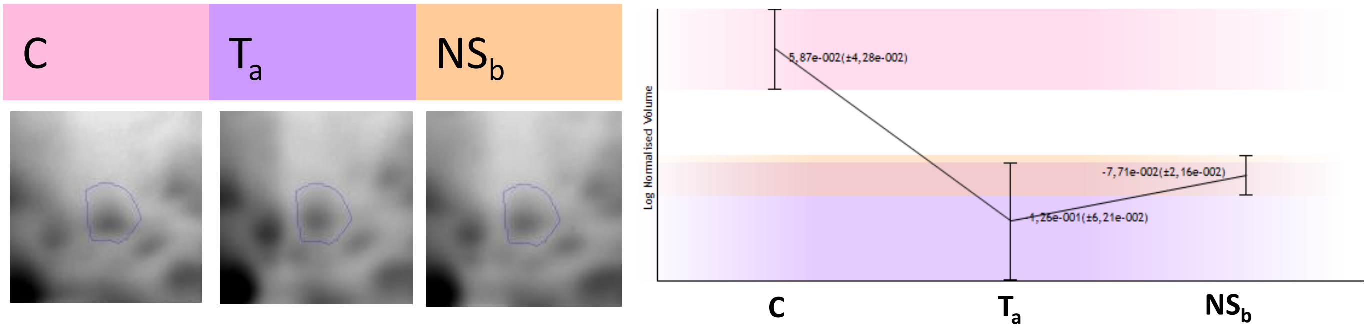

Spot 124- Ger F5

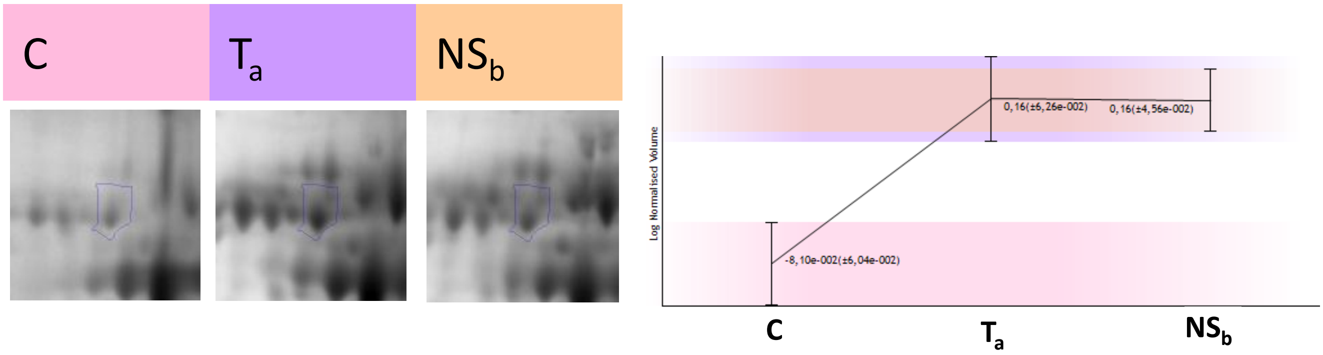

Spot 129- Ger F5

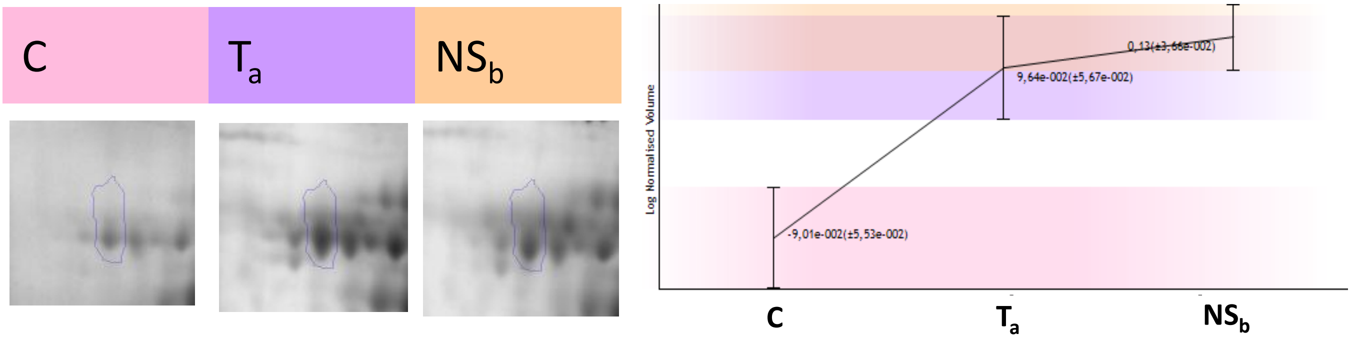

Spot 131- Ger F5

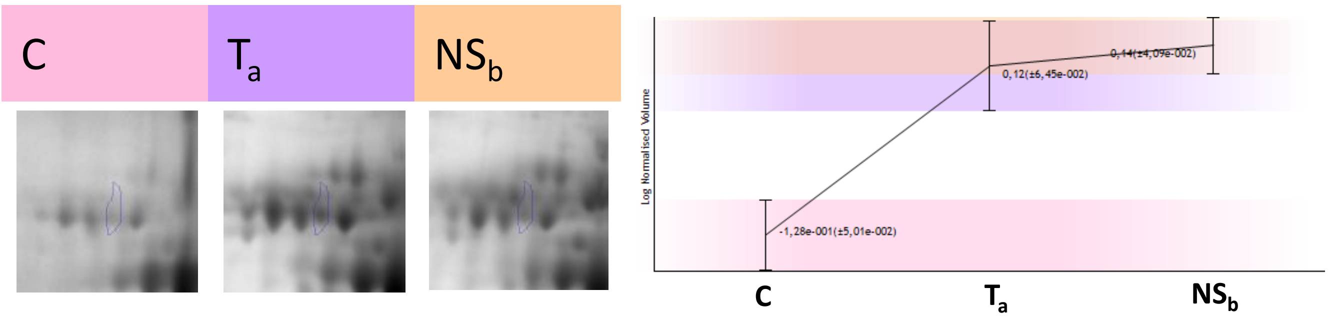

Ger F6

Spot 39- Ger F6

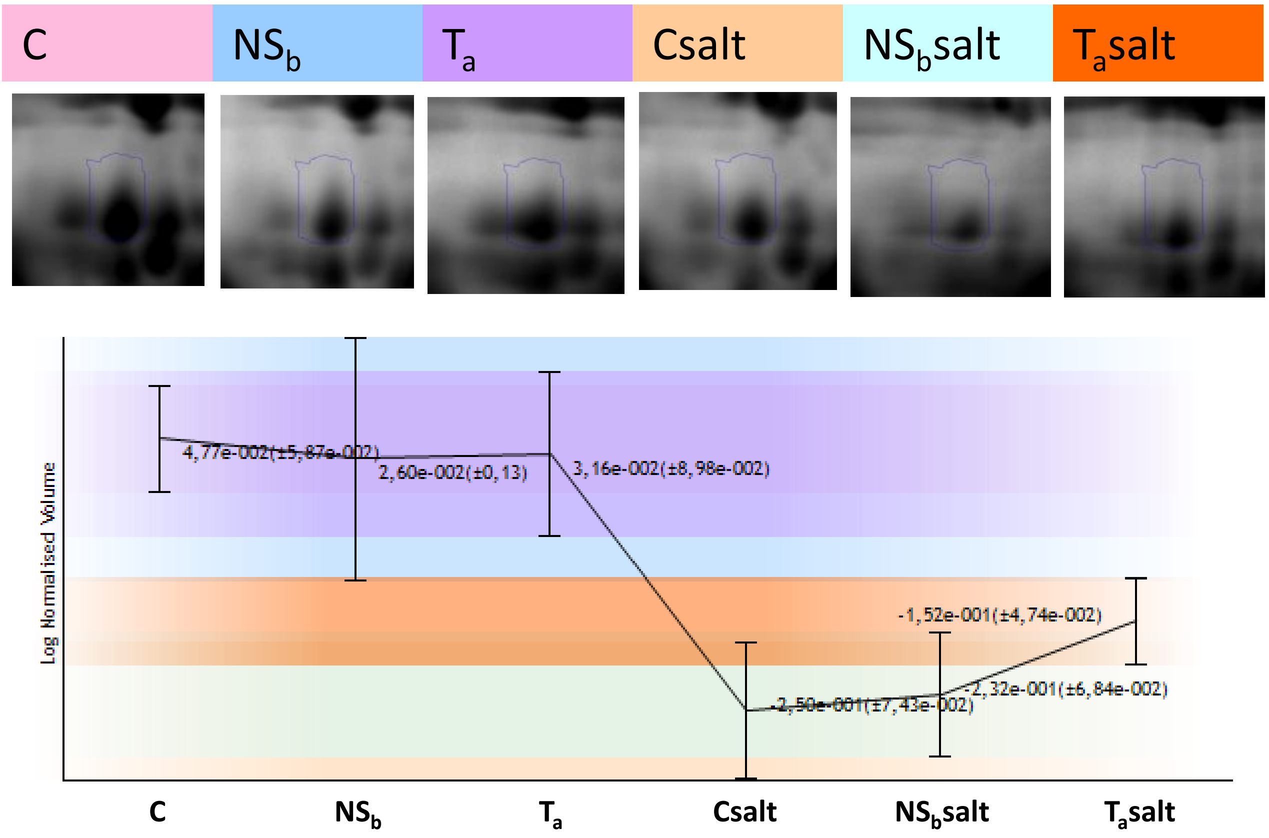

Spot 165- Ger F6

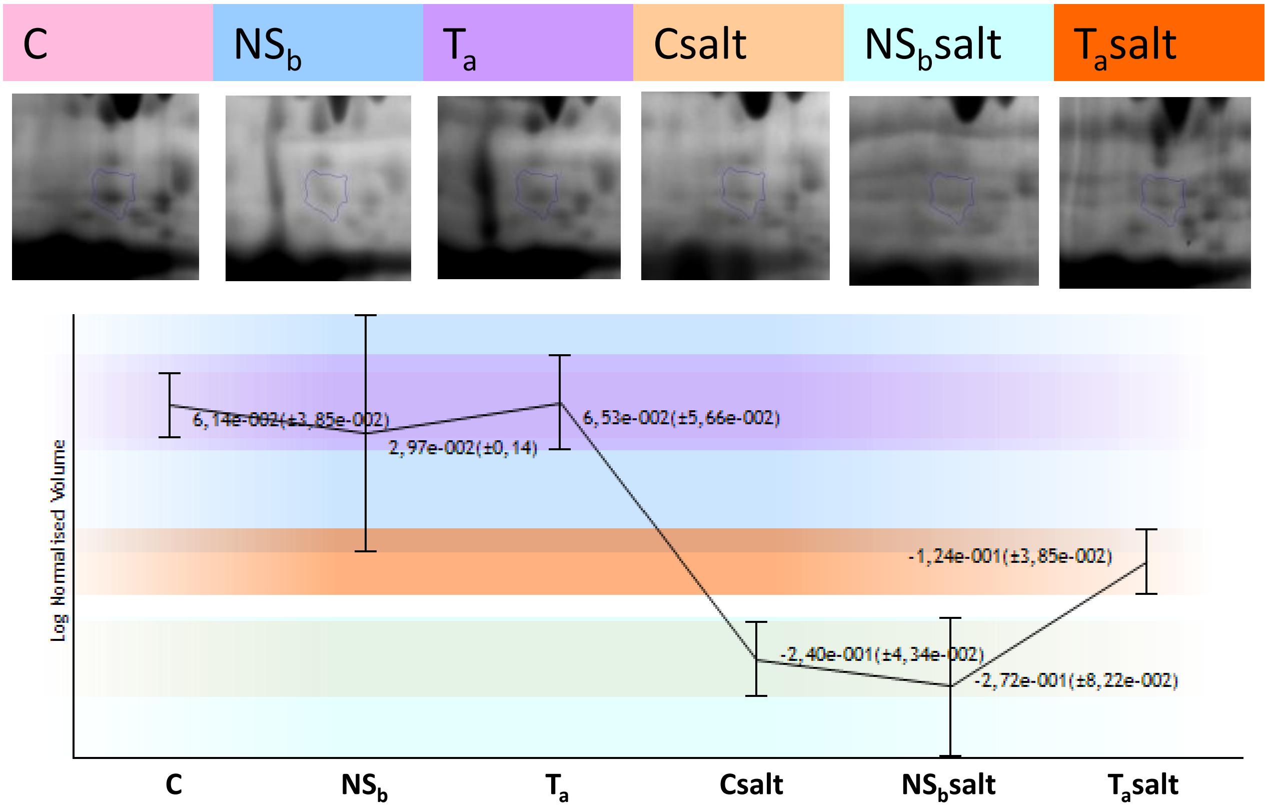

Spot 190- Ger F6

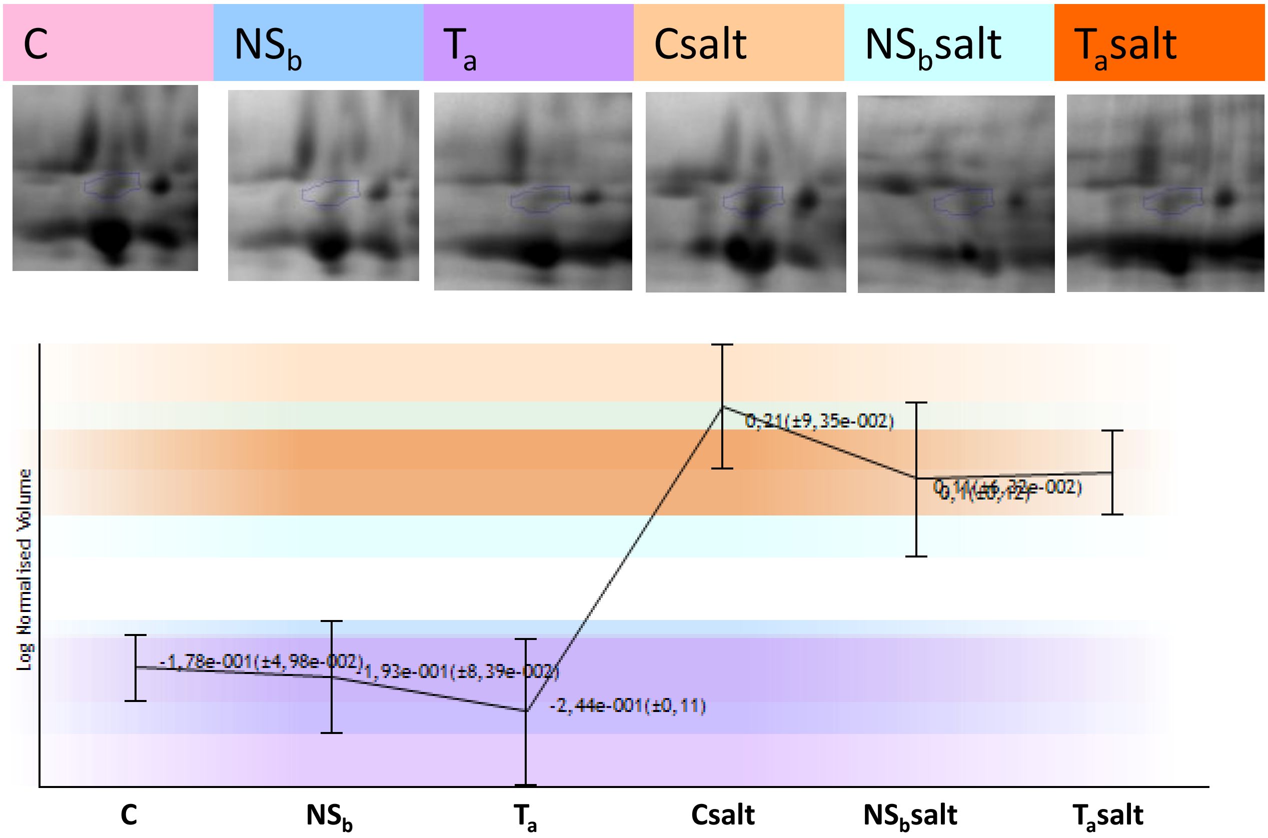

Spot 198- Ger F6

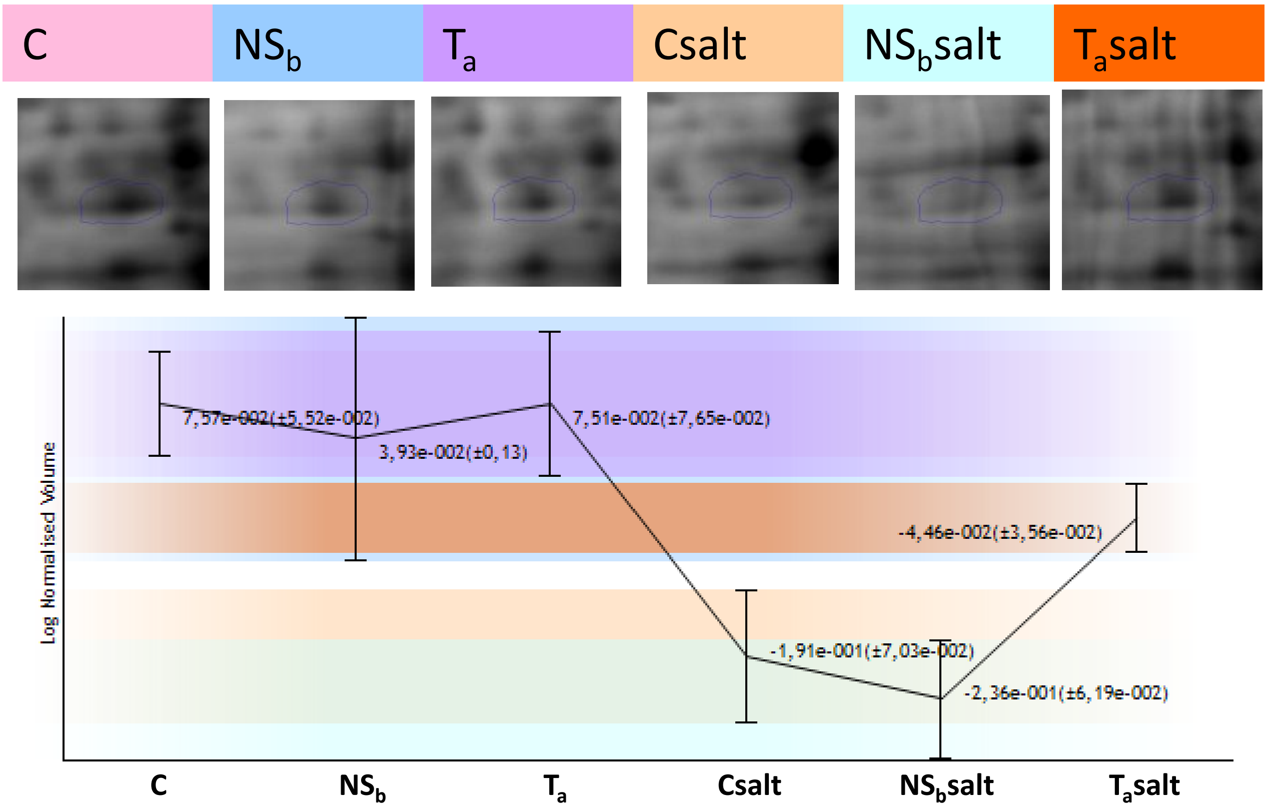

Spot 230- Ger F6

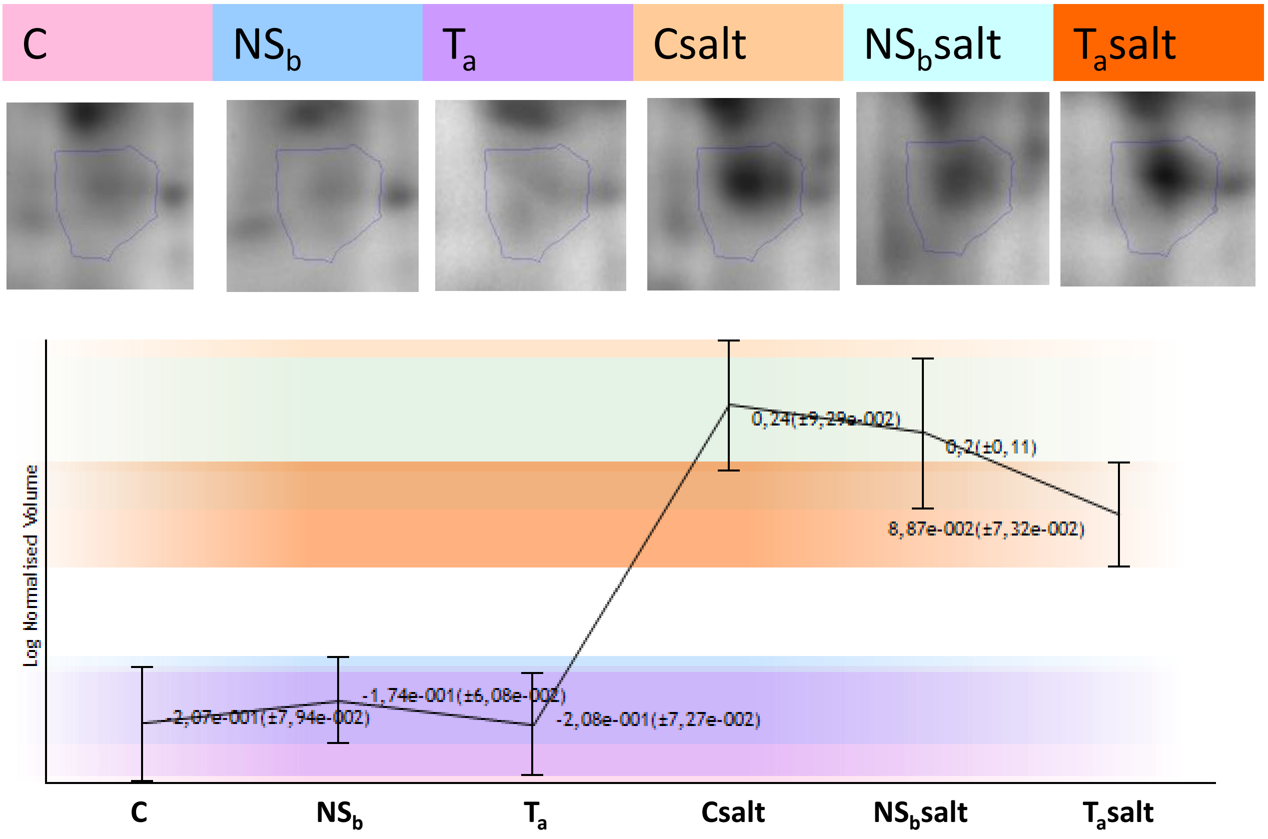

Ger F8

Spot 4- Ger F8

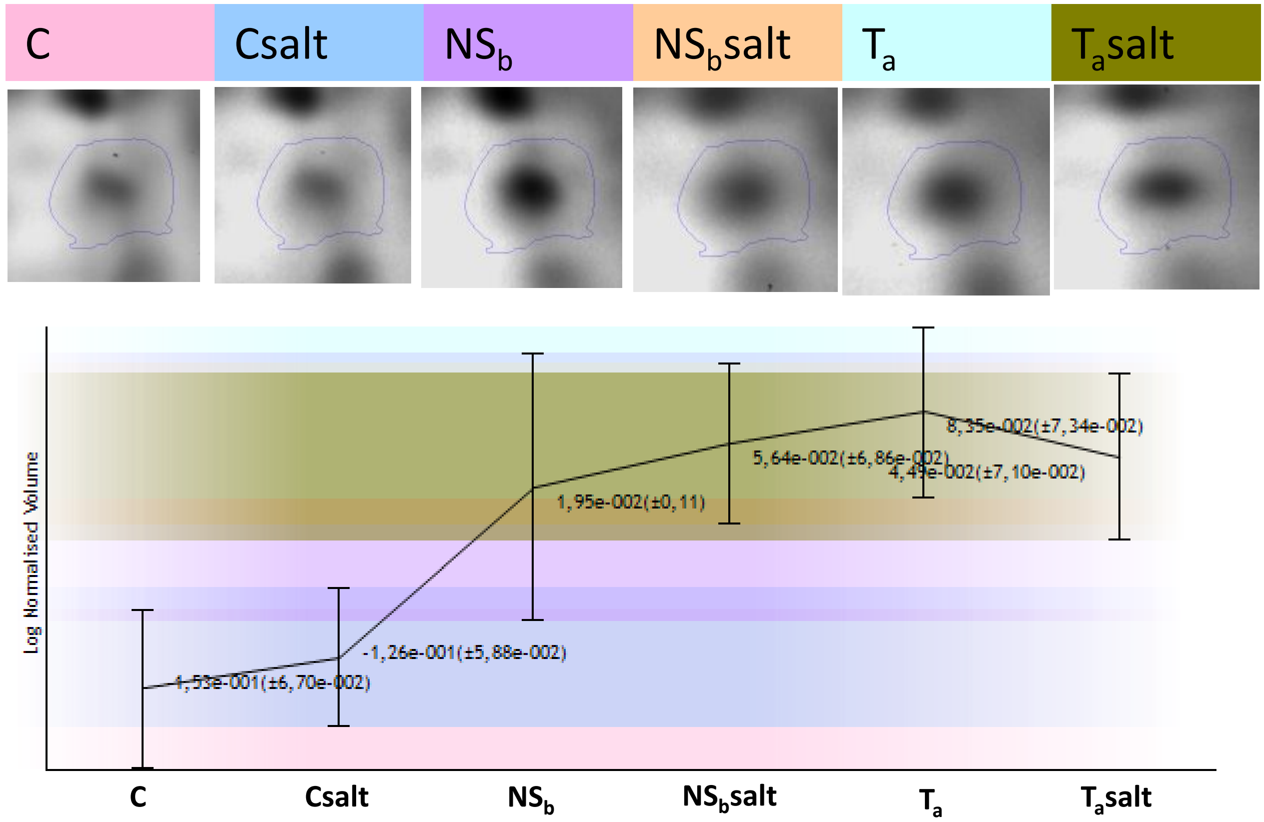

Spot 115- Ger F8

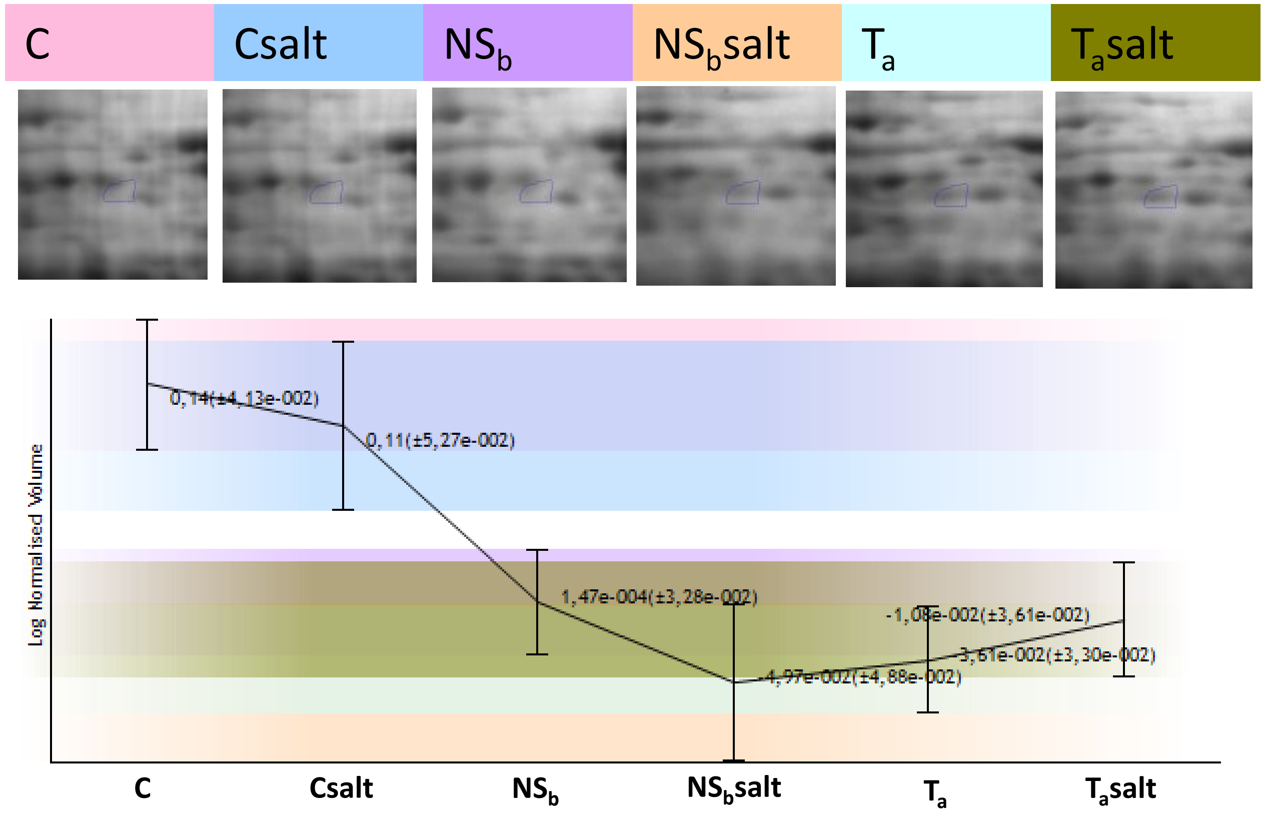

Spot 180- Ger F8

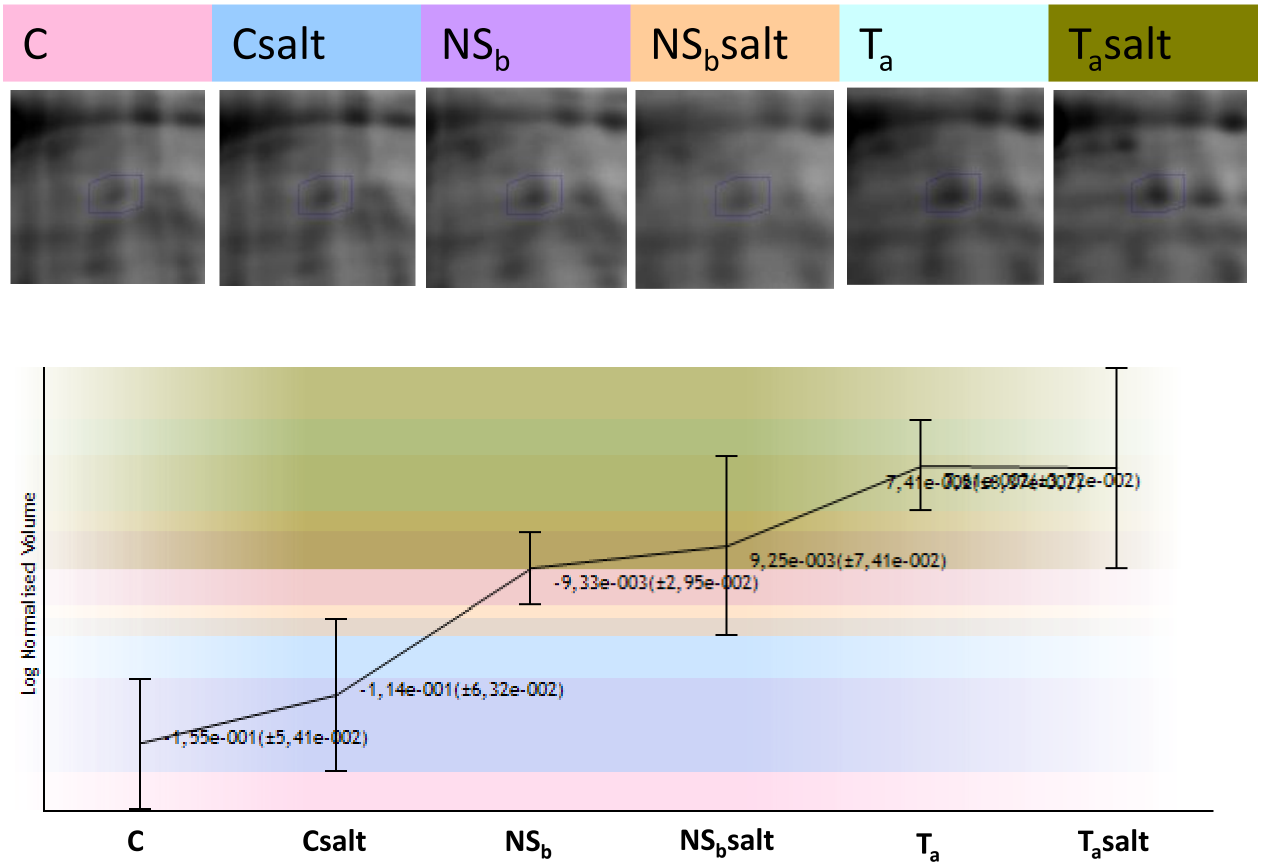

Spot 226- Ger F8

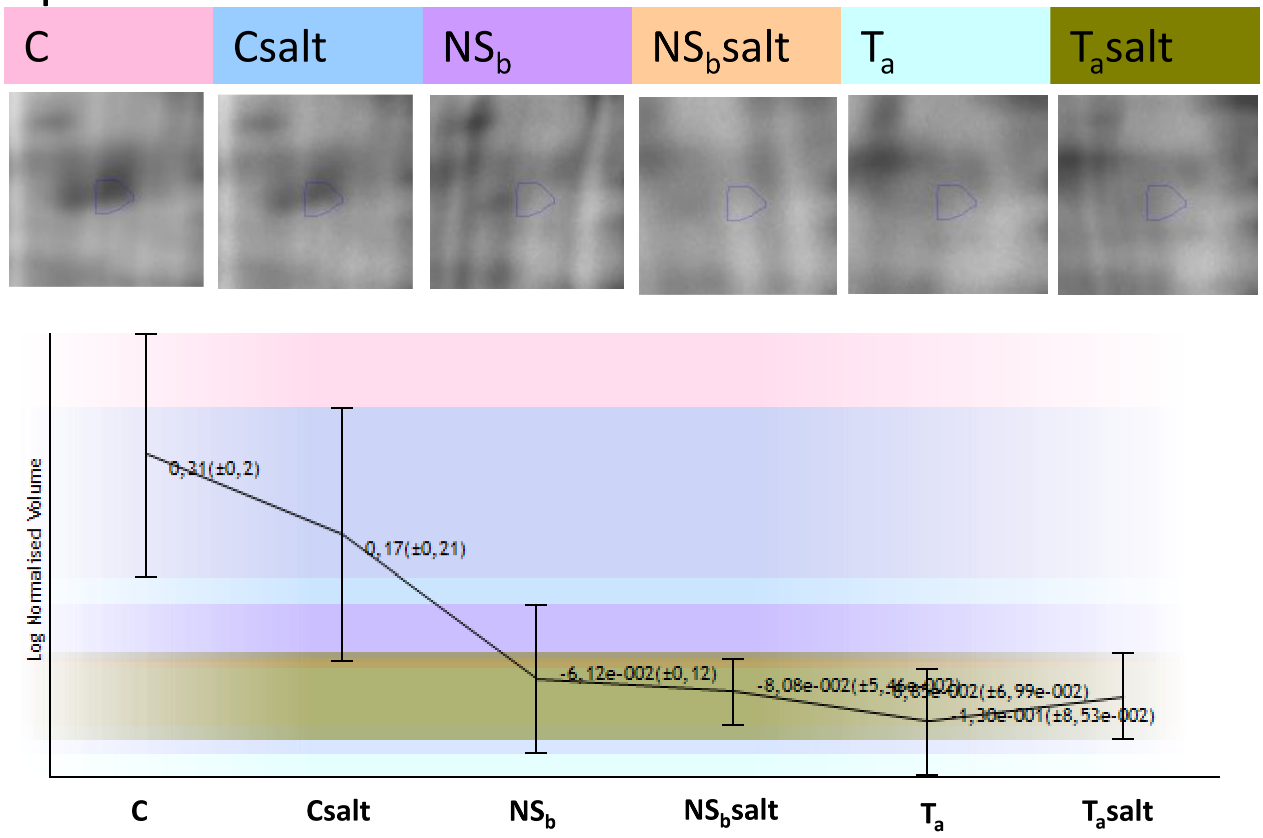

Spot 244- Ger F8

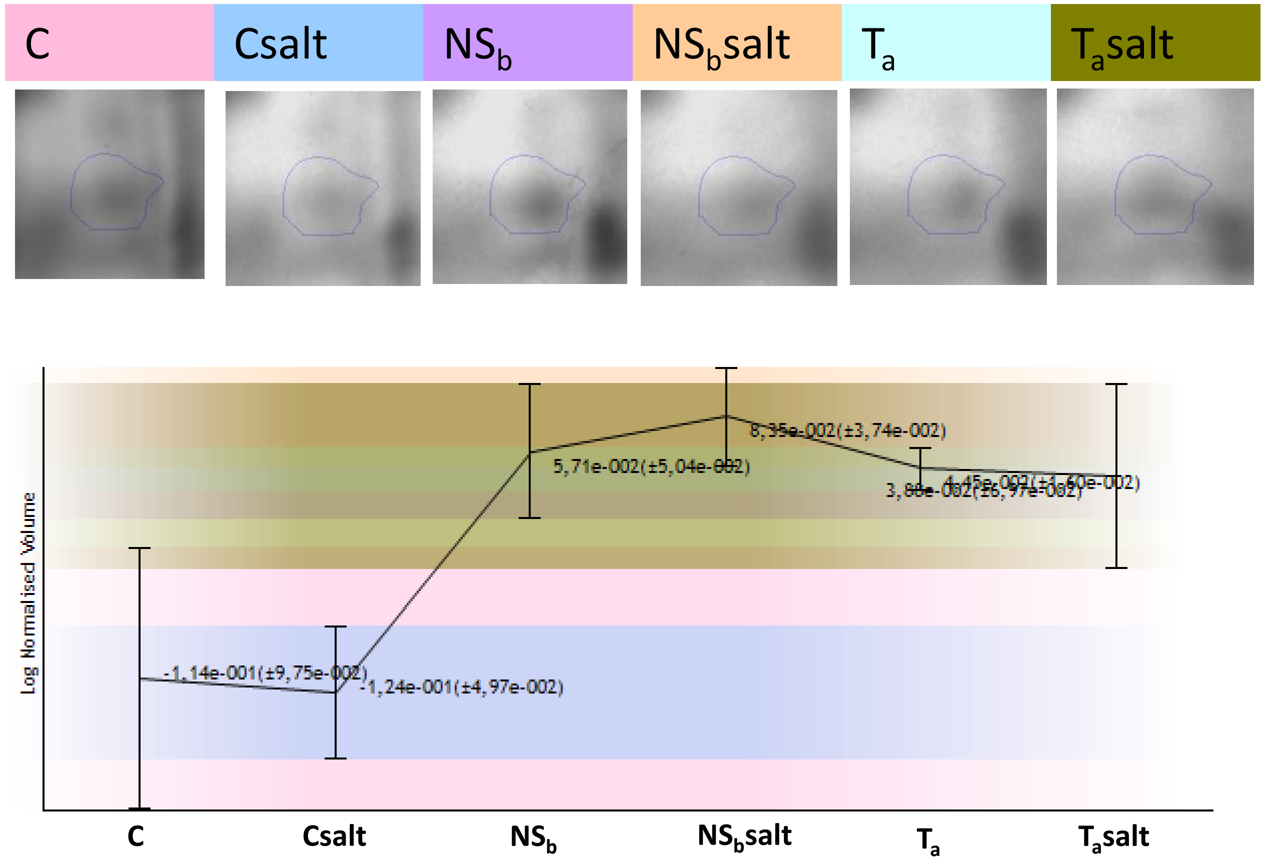

Supporting Table S3- Proteomics and Microarrays relation (salinity assay).

Proteomics				
**Spot nº**	**Most probable protein identification**	**C *vs.* CSalt**	**T *vs.* Tsalt**	**NS *vs.* Nssalt**
**Photosynthesis related**				
**90**	Carbonic anhydrase gi|3345477	-2.3	-1.2	-1.5
**214**		-1.7		
**218**		-2.2	-1.2	-1.3
**207**	Chloroplast 23 kDa polypeptide of photosystem II gi|164375543	+1.8		+1.6
**1**	RuBisCo small chain c gi|77554383	-2.9	-2.5	
**169**	RuBisCo large subunit gi|290874507	-1.9		-1.6
**194**	RuBisCo gi|694137368	-1.6	-1.6	-1.6
**182**	RuBisCO, partial gi|440577954			-1.8
**27**	RuBisCo activase gi|108864712	+1.6		
**95**	Phosphoglycerate kinase, chloroplastic gi|573944490 (Blast)	-1.9		
**Photosynthesis and pentose phosphate pathway**				
**119**	Transketolase 1 gi|55296168	-1.5		
**151**	Transketolase 1 gi|55296168	-1.8		-1.8
**Photorespiration**				
**105**	Phosphoglycolate phosphatase region gi|116310328	-1.6	-1.5	
**183**	Glycine hydroxymethyltransferase gi|31126793	-1.6		-1.4
**Carbohydrate metabolism**				
**195**	Glyceraldehyde-3-phosphate dehydrogenase 2, cytosolic gi|573940046 (Blast)	+1.5	+1.2	+1.2
**196**		+1.6		
**198**	Glyceraldehyde-3-phosphate dehydrogenase B, chloroplastic gi|573923495 (Blast)	-1.8	-1.3	-1.9
**202**	Fructose-bisphosphate aldolase, chloroplastic gi|108864048	-1.6	-1.4	-1.6
**41**	Fructose-bisphosphate aldolase cytoplasmic gi|573915065 (Blast)	+2.3		
**236**		+1.6	+1.3	
**237**		+1.9	+1.3	
**190**	Phosphoglucose isomerase gi|639684	+2.5	+2.3	+2.0
**200**	Fructokinase 1 gi|385145615	-1.6	-1.2	-1.3
**243**	Adenosine diphosphate glucose pyrophosphatase	+1.5		

Microarrays				
**Affymetrix ID**	**Identification**	**C *vs.* Csalt**	**T*vs.* Tsalt**	**NS *vs.* NSsalt**
**Photosynthesis related**				
LOC_Os08g32840	bifunctional monodehydroascorbate reductase and carbonic anhydrasenectarin-3 precursor	+3.0	+3.0	+3.0
LOC_Os11g24140	plastocyanin-like domain containing protein	+6.7	+5.3	+5.2
**Photorespiration**				
**Carbohydrate metabolism**				
LOC_Os03g22120	sucrose synthase	+3.7	+3.5	+5.9
LOC_Os01g13570	phosphoglycerate mutase	+3.6	+3.2	+4.7
LOC_Os03g55090	alpha-glucan phosphorylast isozyme	+2.6	+3.0	+2.9
LOC_Os08g02700	fructose-bisphosphate aldolase isozyme,	+2.5	+1.9	+3.2
LOC_Os08g31980	trehalose-6-phosphate synthase	+2.5	+4.1	+4.6

	gi|21322655			
**Cell Wall polyshacaride metabolism**				
**170**	Glycosyl hydrolase family 38 gi|108864437	-1.5	-1.3	-1.5
**188**	Glycosyl hydrolase family 3 gi|108711094	-1.5	-1.2	-1.2
**189**	Glycosyl hydrolase family 3 gi|108711094	-1.9	-1.2	-1.4
**115**	Beta-D-xylosidase 4 gi|357166259 (Blast)	-1.7		-1.3
**167**	Beta-D-xylosidase 4 gi|573942853	-1.6		-1.3
**219**	RmlD substrate binding domain containing protein gi|108707480	+3.2		+1.7
**TCA/ glyoxylate cycle/ respiratory chain**				
**181**	Pyruvate dehydrogenase E1 component subunit alpha-1, mitochondrial-like gi|573920524	+1.4	+1.3	+1.5
**131**	Aconitate hydratase, cytoplasmatic gi|108706066	-1.7		
**227**	dihydrolipoyllysine-residue succinyltransferase component of 2-oxoglutarate dehydrogenase complex 1, mitochondrial gi|573939599	+2.1	+1.4	+1.5
**165**	Succinate dehydrogenase [ubiquinone] flavoprotein subunit, mitochondrial gi|474445992	-2.0	-1.6	-2.0
**168**	Succinate dehydrogenase [ubiquinone] flavoprotein subunit, mitochondrial gi|573951965 (Blast)	-1.7		-1.5
**Amines metabolism and nitrogen status**				
**148**	5-methyltetrahydropteroyltriglutamate-homocysteine methyltransferase gi|108862990	-1.6		
**149**		-1.7		
**161**		-1.9	-1.2	
**163**	5-methyltetrahydropteroyltriglutamate-homocysteine methyltransferase gi|108862992	-1.8		
**155**		-1.5	+1.3	
**159**		-1.9		-1.4
**201**	Cysteine synthase, chloroplastic/chromoplastic gi|573946673	-1.7	-1.4	-1.6
**204**		+1.8	+1.3	+1.4

**Cell Wall polyshacaride metabolism**				
LOC_Os01g71340	glycosyl hydrolases family 17	+7.8	+7.2	+9.8
LOC_Os01g71830		+6.1	+5.7	+10.4
LOC_Os01g71670		+2.6	+2.2	+3.8
LOC_Os01g71820		+4.2	+3.0	+3.0
LOC_Os10g28120	glycosyl hydrolase	+3.9	+3.5	+4.9
LOC_Os11g47500		+4.4	+4.7	+4.6
LOC_Os11g47600		+2.9	+3.4	+3.3
LOC_Os02g01590		-3.2	-4.1	-2.8
LOC_Os05g15770		+9.7	+10.6	+11.6
LOC_Os07g35560	glucan endo-1,3-beta-glucosidase precursor	+2.3	+5.3	+4.0
LOC_Os09g31430	Os9bglu30 - beta-glucosidase	+3.2	+4.2	+3.3
LOC_Os10g35070	alpha-galactosidase precursor	+2.6	+1.9	+3.3
LOC_Os08g44040	rhamnogalacturonate lyase	-8.4	-5.7	-11.2
LOC_Os07g32630	UDP-glucoronosyl and UDP-glucosyl transferase	-3.0	-2.5	-2.5
LOC_Os02g51930	UDP-Glycosyltransferase family	+3.2	+2.6	+5.0
**TCA/ glyoxylate cycle/ respiratory chain**				
LOC_Os01g41810	cytochrome P450 72A1	+3.6	+3.1	+4.8
LOC_Os10g17260	cytochrome P450	-3.8	-3.4	-3.9
LOC_Os02g09390		+3.1	+3.5	+2.9
LOC_Os04g09920		+3.6	+2.0	+2.1
LOC_Os06g37300		+3.1	+2.3	+3.7
LOC_Os07g11739		+2.7	+2.2	+3.1
LOC_Os08g39730		+3.6	+3.2	+4.4
LOC_Os03g12820	ATP synthase protein 8	+5.9	+4.3	+7.0
LOC_Os07g34520	isocitrate lyase	+2.4	+2.8	+4.3
**Amines metabolism and nitrogen status**				
LOC_Os10g28350	1,2-dihydroxy-3-keto-5-methylthiopentene dioxygenase	+4.3	+3.2	+4.2
LOC_Os03g09910	aminotransferase, classes I and II, domain	+8.5	+11.1	+10.5
LOC_Os11g42510	tyrosine aminotransferase	+3.5	+2.7	+3.4
LOC_Os10g25130	aminotransferase, classes I and II, domain	+3.3	+3.0	+3.2
LOC_Os08g04540	decarboxylase	+2.6	+2.7	+3.3
LOC_Os02g54254	lysine-ketoglutarate reductase/saccharopine dehydrogenase	+4.9	+4.8	+7.1
LOC_Os03g63330	aspartokinase	+5.1	+3.5	+4.2
LOC_Os03g18130	asparagine synthetase	+2.8	+2.7	+3.5

178	Adenosylhomocysteinase-like gi|573961524	-2.2		-1.4
179	Adenosylhomocysteinase-like gi|29367605	-1.6		-1.3
154	Ketol-Acid Reductoisomerase, chain A gi|226887786	-1.7	-1.5	-1.4
209	B0616E02-H0507E05.5 gi|116310889	+1.6		+1.5
173	Ferredoxin--nitrite reductase, chloroplastic gi|75102800	-2.3	-1.3	-1.9
85	S-adenosylmethionine synthase (MAT) gi|3024122	+2.0	+1.3	+1.7
180	S-adenosylmethionine synthase (MAT) gi|17529621	-1.5		
	**Glycine cleavage system**			
39	Aminomethyltransferase, mitochondrial gi|357166054 (Blast)	-2.0	-1.5	-1.8
	**Lipid metabolism**			
113	ATP-citrate synthase beta chain protein 1 gi|357131914	+1.5		
	**Metabolism**			
184	Formate-tetrahydrofolate ligase gi|51536102	-3.3		-1.4
224	NADH-cytochrome b5 reductase gi|514776060 (Blast)	+1.8		+1.3
211	Esterase_lipase region gi|125526544	+2.4	+1.4	+1.7
17	Soluble inorganic pyrophosphatase 1, chloroplastic gi|573922996	-1.7		
220	Oxidoreductase, aldo/keto reductase family gi|413924063	-1.6	-1.5	-1.5
239	Nucleoside diphosphate kinase 4 gi|573946659 (Blast)	+1.8	+1.4	+1.5
210	Pyridoxine 5'-phosphate (PNP) oxidase gi|218199883	+1.8		
	**Modifying, storing and degrading proteins**			
118	DnaK-type molecular chaperone gi|46805772	-1.6	-1.4	
153	Chaperonin 60 subunit beta 1, chloroplastic, gi|573946766	-1.7		-1.3
262	Alpha 4 subunit of 20S proteasome, gi|9186906	+1.6		
91	Proteasome subunit beta type-4-like gi|573957260	-1.9	-1.2	-1.5
104	DSBA-like thioredoxin domain containing protein gi|195648623 (Blast)	+1.7	+1.5	+1.6
65	Thimet oligopeptidase-like gi|573921154 (Blast)	-1.3		-1.5
130	Prolyl oligopeptidase family protein gi|78708638	-1.6		
257	Cytosol alanyl aminopeptidase gi|565830595	-2.1		
255	Peptidyl-prolyl cis-trans isomerase gi|13486733	+1.8		

LOC_Os04g58390	allantoinase	-5.2	-5.3	-5.8
LOC_Os04g40040	copper/topaquinone oxidase family	-2.7	-3.1	-3.2
LOC_Os09g25810	nodulin	-2.4	-3.0	-3.6
LOC_Os06g46740	early nodulin 20 precursor	+6.9	+3.8	+9.0
LOC_Os07g49270	AMP deaminase	+2.6	+2.3	+3.4
LOC_Os04g14690	flavin-containing monooxygenase family protein	+2.3	+5.5	+4.5
LOC_Os07g34570	Putative thiamine biosynthesis protein	+10.9	+13.3	+14.5
LOC_Os11g19840	O-methyltransferase	+11.9	+15.5	+22.8
LOC_Os04g11970	O-methyltransferase	-3.5	-2.4	-2.7
	**Glycine cleavage system**			
	**Lipid metabolism**			
LOC_Os12g37260	lipoxygenase 2.1, chloroplast precursor	+11.9	+14.1	+12.4
LOC_Os02g37654	lecithin:cholesterol acyltransferase	-2.7	-3.2	-3.1
LOC_Os03g52860	lipoxygenase	+4.3	+3.6	+4.8
LOC_Os03g51010	Putative lipase	+2.9	+2.2	+3.1
LOC_Os08g37210	patatin	+6.5	+4.2	+4.7
LOC_Os07g34280	CXE carboxylesterase	+2.7	+3.7	+3.5
LOC_Os04g43200	Peroxygenase	+5.7	+5.3	+9.5
	**Metabolism**			
LOC_Os01g54030	NADP-dependent malic enzyme	+6.4	+5.0	+12.6
LOC_Os12g12590	NADP-dependent oxidoreductase	+3.4	+2.8	+2.1
LOC_Os12g27350	10-deacetylbaccatin III 10-O-acetyltransferase	+10.0	+6.2	+7.5
LOC_Os12g27220	transferase family protein	**+2.6**	**+3.1**	**+3.2**
LOC_Os12g27254		+13.8	+15.3	+8.9
LOC_Os08g02030		+2.6	+2.9	+3.3
	**Modifying, storing and degrading proteins**			
LOC_Os02g32520	Chaperone protein ClpD1, chloroplastic	+3.3	+2.7	+5.6
LOC_Os01g03310	BBTI1 - Bowman-Birk type bran trypsin inhibitor	+5.1	+3.6	+4.9
LOC_Os01g03320	BBTI2 - Bowman-Birk type bran trypsin inhibitor	+3.8	+5.2	+6.3
LOC_Os01g03360	BBTI5 - Bowman-Birk type bran trypsin inhibitor	+3.7	+3.5	+5.1
LOC_Os01g04050	BBTI12 - Bowman-Birk type bran trypsin inhibitor	+3.3	+3.9	+4.3
LOC_Os10g36070	LTPL155 - Protease inhibitor/seed storage/LTP family protein	+3.6	+3.1	+3.6
LOC_Os10g36090	LTPL156 - Protease inhibitor/seed storage/LTP family protein	+3.7	+3.5	+6.7
LOC_Os04g46810	LTPL120 - Protease inhibitor/seed storage/LTP family	-3.1	-3.7	-4.2
LOC_Os10g36100	LTPL157 - Protease inhibitor/seed storage/LTP family protein	+3.5	+2.8	+2.8

	(Blast)			
139	Xaa-Pro aminopeptidase 2 gi|52076499	-1.8		-1.5
245	Immunophilin/FKBP-type peptidyl-prolyl cis-trans isomerase-like gi|34393414	+1.5		
**Signal transduction, stress, defense response, apoptosis**				
84	Root specific pathogenesis-related protein 10 gi|38678114	+2.8	+1.9	+2.1
223	Germin-like protein 1 gi|573912743 (Blast)	+1.8	+1.8	+1.8
6	Abscisic stress ripening protein 2 gi|149391461	+2.1		+1.3
94	Small Ras-related GTP-binding protein gi|16903082	-2.3	-1.4	-1.4
21	Chitinase Chain A, Crystal Structure Of Class I gi|146386456	+6.4	+3.1	+3.4
152	Heat shock protein 90 gi|294717842	+2.2		
246	heat shock protein B, 16.9 kDa gi|312983209	+2.6	+1.4	+1.8
171	hsp70-Hsp90 organizing protein gi|721665923 (Blast)	-2.1	-1.4	-1.5
231	Thaumatin, pathogenesis-related family protein gi|115489698	+1.7	+1.2	+1.4

LOC_Os04g46820	LTPL121 - Protease inhibitor/seed storage/LTP family protein	-3.0	-2.9	-3.3
LOC_Os04g33920	LTPL102 - Protease inhibitor/ seed storage/LTP family protein	+2.9	+3.3	+3.3
LOC_Os03g52390	PIII1 - Proteinase inhibitor II family	+4.3	+3.6	+4.8
LOC_Os03g54130	cysteine protease 1	-7.4	-8.3	-12.5
LOC_Os04g01710	cysteine proteinase At4g11310	+2.9	+3.1	+3.4
LOC_Os09g39070	thiol protease SEN102	-2.8	-2.4	-3.7
LOC_Os09g19800	aminopeptidase	+7.1	+4.8	+6.0
LOC_Os01g60730	RING-H2 finger protein	+2.3	+3.6	+3.3
LOC_Os11g34460	OsFBO10 - F-box and other domain containing protein	+2.4	+5.7	+3.4
LOC_Os01g58280	Subtilisin-like serine protease	+3.8	+2.7	+6.2
LOC_Os01g42860	Subitilisin-chymotrypsin inhibitor	+16.8	+12.6	+31.7
LOC_Os12g36240	Subtilisin-chymotrypsin inhibitor CI-1B	+6.2	+6.4	+10.8
LOC_Os04g03810	OsSub38 - Putative Subtilisin	+2.5	+4.2	+3.2
LOC_Os01g06740	ribosome inactivating protein	+11.5	+9.5	+12.9
LOC_Os11g08100	eukaryotic aspartyl protease	+2.4	+3.0	+3.1
LOC_Os06g16640	carboxyl-terminal peptidase	+3.1	+2.9	+3.2
**Signal transduction, stress, defense response, apoptosis**				
LOC_Os01g14550	pathogen-related protein	+2.3	+3.3	+3.0
LOC_Os12g36880	pathogenesis-related Bet v I family protein	+4.2	+2.7	+4.9
LOC_Os01g32780	universal stress protein domain containing protein	+4.7	+4.3	+5.5
LOC_Os02g47840	universal stress protein domain containing protein	+2.4	+2.1	+3.3
LOC_Os10g39680	CHIT14 - Chitinase family protein precursor	+3.6	+3.1	+4.8
LOC_Os04g41620	CHIT2 - Chitinase family protein precursor	+6.4	+5.6	+5.8
LOC_Os04g41680	CHIT3 - Chitinase family protein precursor	+3.1	+3.2	+3.9
LOC_Os05g33140	CHIT5 - Chitinase family protein precursor	+3.0	+2.2	+3.1
LOC_Os06g51050	CHIT7 - Chitinase family protein precursor	+6.4	+6.8	+8.0
LOC_Os06g51060	CHIT8 - Chitinase family protein precursor	+4.6	+4.1	+5.1
LOC_Os04g41640	HEV2 - Hevein family protein precursor	+3.2	+2.8	+3.2
LOC_Os11g37940	WIP2 - Wound-induced protein precursor	+5.5	+4.2	+5.6
LOC_Os11g37950	WIP3 - Wound-induced protein precursor	+5.4	+3.4	+6.6
LOC_Os11g37970	WIP5 - Wound-induced protein precursor	+5.6	+4.9	+7.2
LOC_Os04g01740	Heat shock protein	+2.8	+2.2	+3.2
LOC_Os03g15960	hsp20/alpha crystallin family protein	+3.1	+2.4	+3.1
LOC_Os03g16920	Heat shock cognate 70 kDa protein	+2.7	+2.3	+3.7
LOC_Os12g43450	Thaumatin	+4.0	+3.9	+4.5
LOC_Os12g43380		+22.7	+7.4	+23.3

249	SalT gene product gi|2072553	+2.3		+2.2
250		+2.3		
186	Monocopper oxidase-like protein SKU5-like gi|573959489 (Blast)	-1.6		-1.3

LOC_Os12g43410		+3.1	+3.4	+2.7
LOC_Os03g46070		+4.1	+3.0	+4.0
LOC_Os11g26780	dehydrin	+6.0	+4.1	+14.4
LOC_Os11g26790		+11.2	+6.1	+12.7
LOC_Os12g38170	osmotin	+3.1	+2.7	+3.8
LOC_Os03g17790	OsRCI2-5 - Putative low temperature and salt responsive protein	+4.2	+3.6	+5.4
LOC_Os01g28450	SCP-like extracellular protein	+21.9	+7.0	+23.9
LOC_Os01g28500		+5.3	+4.0	+4.4
LOC_Os10g11500		+3.9	+3.2	+2.7
LOC_Os07g03279		+3.7	+3.2	+2.9
LOC_Os07g03319		+8.6	+8.5	+10.3
LOC_Os07g03368		+3.6	+3.2	+2.7
LOC_Os07g03409		+6.3	+5.3	+6.0
LOC_Os07g03458		+3.7	+3.1	+3.0
LOC_Os07g03499		+6.3	+5.3	+6.1
LOC_Os07g03730		+12.3	+6.8	+9.2
LOC_Os07g03288		+24.7	+13.2	+19.7
LOC_Os07g03377		+24.7	+13.2	+19.7
LOC_Os07g03467		+24.5	+13.6	+18.4
LOC_Os07g25050	thionin-like peptide	+2.6	+3.5	+2.4
LOC_Os07g25060		+3.5	+4.7	+3.1
LOC_Os07g24830		+5.0	+7.1	+4.3
LOC_Os10g09850	EF hand family protein	+3.6	+3.1	+5.0
LOC_Os01g26270	OsWAK7 - OsWAK receptor-like protein kinase	-3.0	-2.4	-2.8
LOC_Os08g10250	SHR5-receptor-like kinase	-4.6	-3.9	-3.7
LOC_Os08g10300	SHR5-receptor-like kinase	+2.3	+3.2	+2.7
LOC_Os07g43570	DUF26 kinase	+3.2	+4.7	+5.5
LOC_Os11g31540	Brassinosteroid insensitive 1-associated receptor kinase 1	+3.2	+2.5	+4.5
LOC_Os01g62900	amino acid kinase	+3.5	+3.4	+5.1
LOC_Os02g02780	protein kinase family protein	+4.0	+6.8	+5.0
LOC_Os04g56430	cysteine-rich receptor-like protein kinase	+7.8	+5.6	+9.7
LOC_Os07g36544	serine/threonine-protein kinase receptor	+2.4	+2.6	+3.9
LOC_Os06g48300	protein phosphatase 2C	+10.5	+12.5	+20.6
LOC_Os10g41980	RALFL26 - Rapid ALkalinization Factor RALF family	+9.5	+5.9	+8.4
LOC_Os06g29730	RALFL28 - Rapid ALkalinization Factor RALF	+6.6	+6.1	+9.3
LOC_Os11g02240	CAMK includes calcium/calmodulin dependent protein	+5.7	+4.2	+7.3
LOC_Os01g24710	jacalin-like lectin domain containing protein	+3.3	+3.5	+4.6
LOC_Os12g14440		+97.0	+86.7	+94.7
LOC_Os12g09700		+3.9	+3.8	+3.9
LOC_Os04g38360	wound/stress protein	+7.1	+6.1	+4.6
LOC_Os05g46480	late embryogenesis abundant protein, group 3	+10.8	+5.9	+16.6

**Glyoxalase system**				
96	Lactoylglutathione lyase (Glioxalase I) gi|341870587	-1.2	-1.4	-1.6
**Phenylpropanoids metabolism and related proteins**				
160	Phenylalanine/tyrosine ammonia-lyase gi|514715067	-4.4	-2.2	-2.3
164	Phenylalanine ammonia-lyase gi|573919867 (Blast)	-2.0	-1.5	-1.7
185				-1.6
**Terpene biosynthesis**				
**Antioxidation**				
3	Superoxide dismutase [Cu-Zn] gi|115473931	+2.0	+1.6	+1.8
247	Superoxide dismutase [Cu-Zn] gi|42408425	+1.9		+1.4
5	Superoxide dismutase gi|538430	+1.7		
213	Ascorbate_peroxidase gi|115458488	+1.5		
205	L-ascorbate peroxidase 1 gi|158512874 (Blast)	+1.8	+1.4	+1.4
208		+1.6	+1.2	+1.3
16	IN2-1 protein (Glutathione S-transferase GSTZ5) gi|108707532	-2.0		
241	GSH-dependent dehydroascorbate reductase 1 gi|6939839	+1.5	+1.3	+1.4
244	Peroxiredoxin-2F, mitochondrial-like gi|573912657	+1.9	+1.3	+1.7

LOC_Os03g47610	thiamine biosynthesis protein thiC	+3.8	+5.0	+7.0
LOC_Os11g37200	transmembrane BAX inhibitor motif-containing protein	+3.2	+2.3	+3.0
LOC_Os12g29400	GRAM domain containing protein	+2.8	+3.0	+4.8
LOC_Os09g08130	indole-3-glycerol phosphate synthase	+3.0	+3.3	+4.0
LOC_Os05g39250	Putative cold regulated protein	+4.7	+2.9	+4.8
LOC_Os03g48750	Germin-like protein 3-3	+6.8	+8.1	+10.6
LOC_Os03g48780	Germin-like protein 3-6	+7.7	+6.7	+7.1
LOC_Os06g19300	cadmium tolerance factor	+2.7	+3.2	+2.4
LOC_Os09g28160	Putative phosphate transport protein	+2.4	+3.1	+4.2
LOC_Os03g57640	gibberellin receptor GID1L2	+5.6	+6.8	+8.9
**Glyoxalase system**				
**Phenylpropanoids metabolism and related proteins**				
LOC_Os01g58100	polyphenol oxidase	+3.5	+4.1	+4.3
LOC_Os10g18870	dirigent	-19.0	-17.0	-17.2
LOC_Os10g18820		-3.4	-3.5	-3.3
LOC_Os01g13610	isoflavone reductase homolog IRL	+3.3	+3.3	+3.0
LOC_Os10g40934	flavonol synthase/flavanone 3-hydroxylase	+2.4	+2.8	+3.7
LOC_Os12g17160	flavonol sulfotransferase	+2.7	+2.1	+3.0
LOC_Os07g11440	chalcone synthase	+4.4	+4.0	+2.8
LOC_Os09g17560	Flavonoid 7-O-methyltransferase-like	+2.2	+2.5	+3.4
**Terpene biosynthesis**				
LOC_Os04g01810	terpene synthase	+2.4	+1.7	+3.4
LOC_Os04g27430		+11.9	+10.2	+12.4
LOC_Os04g27670		+4.0	+4.0	+6.8
LOC_Os08g07080		+4.3	+3.5	+3.3
LOC_Os08g04500		-2.3	-3.4	-3.2
LOC_Os04g10060	ent-kaurene synthase	+2.7	+3.1	+3.6
LOC_Os06g47130	Putative CLB1 protein	+3.3	+3.0	+4.3
**Antioxidation**				
LOC_Os02g53400	thioredoxin	+3.3	+5.7	+4.8
LOC_Os01g22249	Peroxidase precursor	-3.2	-1.9	-3.1
LOC_Os03g25300		-2.7	-3.2	-4.8
LOC_Os06g35520		+2.2	+3.2	+2.1
LOC_Os07g01410		-3.6	-3.3	-3.6
LOC_Os07g01420		-2.5	-2.7	-3.4

	(Blast)			
**Cell structure and biogenesis**				
233	40S ribosomal protein S3-3-like gi|573953785 (Blast)	+2.1	+1.3	+1.7
229	40S ribosomal protein S5-like gi|573961547 (Blast)			+1.6
230	40S ribosomal protein S7-like gi|514816665	+2.8	+2.0	+2.4
226	60S acidic ribosomal protein P0-like gi|573953644	+2.7	+1.5	+1.6
4	Actin-depolymerizing factor 4 gi|573926554 (Blast)	+2.0		+1.3
206	Ribosome-recycling factor, chloroplastic-like gi|573952885 (Blast)	+1.3	+1.3	+1.5
147	Cell division control protein 48-A gi|15232776	-1.7		
199	Alpha tubulin-1D gi|90289618	-1.5	-1.4	
59	Alanyl-tRNA synthetase, mitochondrial gi|110288715	-1.7		
134	Elongation factor gi|119395218	-2.5		-1.9
133	Elongation factor gi|119395216	-3.0		-1.7
**Transport**				
102	Mitochondrial outer membrane protein porin 1 gi|573956673	+2.2		
232	Porin-like protein gi|115451401	+3.6	+1.7	+2.1

**Cell structure and biogenesis**				
LOC_Os11g32880	DEAD-box ATP-dependent RNA helicase	+4.9	+4.2	+6.6
LOC_Os01g06310	glycine-rich cell wall structural protein	+4.6	+3.2	+4.2
LOC_Os09g29710	beta-expansin	+3.0	+2.5	+5.3
LOC_Os03g60580	actin-depolymerizing factor	+9.3	+10.2	+15.4
**Transport**				
LOC_Os01g43460	C4-dicarboxylate transporter/malic acid transport protein	+2.8	+2.6	+3.8
LOC_Os01g36720	Probable high-affinity nitrate transporter 2.4	**-5.2**	**-5.4**	**-8.5**
LOC_Os02g02170	High-affinity nitrate transporter 2.1	**-9.9**	**-12.0**	**-8.8**
LOC_Os02g38230	high affinity nitrate transporter	**-5.8**	**-7.0**	**-6.1**
LOC_Os10g20470	MATE efflux family protein	+2.3	+2.4	+4.1
LOC_Os04g30490	MATE efflux family protein	+2.7	+3.2	+2.5
LOC_Os10g30770	inorganic phosphate transporter	+5.6	+2.2	+4.9
LOC_Os04g10690	inorganic phosphate transporter	+4.4	+2.7	+5.6
LOC_Os04g51830	OsHKT1	-3.6	-1.9	-3.6
LOC_Os06g48810	OsHKT2 1 - Na+ transporter	-7.0	-4.5	-6.8
LOC_Os10g21590	transporter family protein	+3.1	+5.1	+5.3
LOC_Os04g13210	multidrug resistance-associated protein	+3.4	+3.8	+3.9
LOC_Os02g03900	metal transporter Nramp6	+3.8	+3.3	+2.7
LOC_Os01g50100	ABC transporter, ATP-binding protein	+3.1	+5.0	+4.3
LOC_Os08g29570	ABC transporter G family member 44	+2.4	+2.1	+3.4
LOC_Os07g18874	ABC transporter family protein-like	-3.4	-4.5	-3.4
LOC_Os07g33780	ABC transporter G family member 43	+2.4	+2.3	+3.0
LOC_Os03g11900	transporter family protein	+2.3	+2.4	+3.4
LOC_Os02g44080	aquaporin protein	-3.1	-3.9	-4.6
LOC_Os04g44060	aquaporin protein	-2.7	-3.1	-2.8
LOC_Os04g38680	transmembrane amino acid transporter protein	+2.7	+1.9	+3.2
LOC_Os01g50460	Bidirectional sugar transporter SWEET2b	-3.8	-4.2	-4.5
LOC_Os12g33300	integral membrane protein DUF6 containing protein	+4.8	+5.8	+5.6
LOC_Os12g31860	ureide permease	+4.6	+4.8	+5.0
LOC_Os02g09810	Putative amino acid transporter A1	+3.7	+3.9	+7.2
LOC_Os09g31478	auxin efflux carrier component	+3.7	+4.7	+5.2
LOC_Os09g29660	white-brown complex homolog protein 11	+2.6	+3.0	+3.3
LOC_Os06g03540	oligopeptide transporter	-4.0	-2.8	-3.9

**Control of gene expression**				
**222**	Methionine adenosyltransferase 2 gi|75308025	+3.0		
**259**	Eukaryotic translation initiation factor 3 subunit A gi|514781216 (Blast)	+3.1		+1.5
**221**	chloroplast stem-loop binding protein of 41 kDa b, chloroplastic-like gi|573963758	-1.4	-1.3	-1.6
**RNA binding**				
**Transcription factors**				
**Unknown**				
**254**	Expressed protein gi|108707969	+1.6		
**82**	NON-significant identification	-1.7	-1.6	-1.7
**125**	NON-significant identification	-2.1		-1.8
**135**	NON-significant identification			-1.5
**144**	NON-significant identification	+1.7		
**145**	NON-significant identification	-1.5		
**146**	NON-significant identification	-1.8		
**150**	NON-significant identification	-1.9	-1.2	
**156**	NON-significant identification	-1.8		-1.6
**157**	NON-significant identification	-1.6		
**158**	NON-significant identification	-2.5		-1.9
**162**	NON-significant identification	-3.2	-1.6	-2.0
**166**	NON-significant identification	-2.0		
**172**	NON-significant identification	-2.1		-1.4
**174**	NON-significant identification	-2.8		-1.6
**175**	NON-significant identification	-1.5		

LOC_Os06g07200	syntaxin	+2.4	+3.0	+2.5
**Control of gene expression**				
LOC_Os05g31020	peptide chain release factor subunit 1	+3.8	+3.1	+4.3
LOC_Os02g36590	CPuORF19 - conserved peptide uORF	-4.0	-3.0	-3.9
**RNA binding**				
LOC_Os02g33610	Putative metallo beta subunit lactamase	+2.8	+3.2	+4.7
LOC_Os09g36680	Drought-induced S-like ribonuclease	+47.8	+39.7	+26.0
LOC_Os09g36700	Putative RNase S-like protein	+16.5	+17.7	+11.8
**Transcription factors**				
LOC_Os01g01840	helix-loop-helix DNA-binding domain containing protein	+7.1	+5.1	+6.3
LOC_Os11g02540	OsWRKY50 - Superfamily of TFs having WRKY and zinc finger domains	+6.9	+8.3	+8.1
LOC_Os12g02450	OsWRKY64 - Superfamily of TFs having WRKY and zinc finger domains	+5.6	+5.8	+6.0
LOC_Os12g02470	OsWRKY65 - Superfamily of TFs having WRKY and zinc finger domains	+7.0	+7.5	+6.0
LOC_Os05g50340	MYB family transcription factor	-3.1	-4.5	-4.1
LOC_Os06g45140	bZIP family transcription factor	-2.9	-2.7	-3.2
LOC_Os05g45410	Heat stress transcription factor A-4d	+2.0	+1.8	+3.1
**Unknown**				
LOC_Os05g49300	Putative iron-sulfur cluster assembly complex protein	+3.5	+2.4	+3.4
LOC_Os01g06890	leucine-rich repeat family protein	+4.7	+2.1	+4.9
LOC_Os10g34930	Putative basic secretory protein	+3.0	+2.7	+3.1
LOC_Os10g05820	POEI5 - Pollen Ole e I allergen and extensin family protein	+2.2	+2.8	+3.2
LOC_Os11g45990	von Willebrand factor type A domain containing protein	+4.1	+4.2	+4.9
LOC_Os11g46000	von Willebrand factor type A domain containing protein	+3.6	+3.1	+3.0
LOC_Os06g38080	von Willebrand factor type A domain containing protein	+3.0	+2.8	+2.2
LOC_Os04g51796	DNA repair ATPase-related	+2.8	+2.3	+3.2
LOC_Os05g28770	GCRP9 - Glycine and cysteine rich family protein	-2.3	-2.1	-3.3
LOC_Os05g29710	RING-H2 finger protein	+2.5	+3.2	+3.5
LOC_Os05g36310	zinc finger, C3HC4 type domain containing protein	+2.9	+2.6	+3.1
LOC_Os03g28990	zinc finger family protein	+2.4	+4.2	+2.9
LOC_Os07g09420	ATPase	+2.8	+2.3	+3.4
LOC_Os07g48800	VQ domain containing protein	+3.9	+3.4	+3.5
LOC_Os08g30020	membrane protein	-3.9	-3.5	-3.7
LOC_Os09g31380	jmjC domain-containing protein 5	+2.2	+4.0	+2.7

**176**	NON-significant identification	+1.6		
**177**	NON-significant identification	-1.7		-1.2
**187**	NON-significant identification	-2.3		
**191**	NOT possible to pick from the gel	+1.6	+1.4	
**192**	NOT possible to pick from the gel	+2.0	+1.5	+1.6
**193**	NOT possible to pick from the gel	+1.6		
**197**	NON-significant identification	+1.7		
**203**	NON-significant identification	+1.7	+1.4	+1.4
**212**	NON-significant identification	+1.8	+1.6	+1.6
**215**	NON-significant identification	+1.4		+1.6
**216**	NON-significant identification	+1.3		+1.5
**217**	NON-significant identification	+2.6	+1.7	+1.7
**225**	NOT possible to pick from the gel	+2.5	+1.3	+1.5
**228**	NON-significant identification	+2.8		+2.0
**234**	NOT possible to pick from the gel	+1.8	+1.2	+1.2
**235**	NON-significant identification	+1.7		
**238**	NON-significant identification	+1.9		
**240**	NON-significant identification	+1.6		
**242**	NON-significant identification			+1.6
**248**	NOT possible to pick from the gel	+1.6		+1.5
**251**	NOT possible to pick from the gel	+1.6		
**252**	NOT possible to pick from the gel	+1.8		
**253**	NOT possible to pick from the gel	+2.3	+1.7	+1.7
**256**	NON-significant identification	+1.5	+1.3	+1.4
**258**	NOT possible to pick from the gel	+1.9	+1.2	+1.5
**260**	NOT possible to pick from the gel	+2.9		+2.0
**261**	NON-significant identification		-1.5	

LOC_Os01g67320	expressed protein	-2.7	-3.9	-2.6
Os01g0386500	Os01g0386500 mRNA	+20.0	+13.9	+21.8
Os01g0661800	Os01g0661800 mRNA	+4.7	+3.0	+5.2
Os10g0569600	Os10g0569600 mRNA	+4.5	+4.1	+3.5
Os10g0450900	Os10g0450900 mRNA	+4.1	+3.7	+4.5
Os01g0740700	Os01g0740700 mRNA	+2.4	+2.3	+3.4
LOC_Os11g10590	hypothetical protein	+12.0	+10.7	+10.0
LOC_Os11g13750	expressed protein	-4.5	-3.0	-5.5
LOC_Os01g71624	expressed protein	+11.6	+10.1	+18.5
Os11g0303800	Os11g0303800 mRNA	+7.0	+8.6	+11.9
Os12g0257600	Os12g0257600 mRNA	+10.4	+8.5	+12.7
Os12g0431700	Os12g0431700 mRNA	+3.5	+4.7	+4.0
Os12g0440300	Os12g0440300 mRNA	+3.4	+2.0	+3.1
LOC_Os12g08700	expressed protein	+2.6	+2.2	+3.2
LOC_Os12g11990	expressed protein	+2.5	+3.5	+2.5
Os04g0650700	Os04g0650700 mRNA	-3.0	-4.9	-4.2
LOC_Os05g49940	expressed protein	+2.3	+2.1	+3.1
LOC_Os05g29160	hypothetical protein	+2.5	+2.6	+3.8
LOC_Os05g29680	hypothetical protein	+3.9	+4.8	+3.7
LOC_Os06g04480	expressed protein	+3.3	+2.3	+3.5
LOC_Os06g28630	expressed protein	-5.2	-4.0	-6.7
LOC_Os06g39120	expressed protein	+8.5	+8.5	+7.7
LOC_Os06g05420	expressed protein	+3.6	+4.7	+5.6
LOC_Os12g32610	expressed protein	+3.0	+3.2	+4.0
Os06g0199100	Os06g0199100 mRNA	+3.0	+2.7	+6.1
LOC_Os07g19540	expressed protein	+4.7	+4.2	+5.4
Os07g0431000	Os07g0431000 mRNA	+6.8	+11.7	+8.4
Os12g0222000	Os12g0222000 mRNA	+7.1	+4.8	+8.6
Os07g0418600	Os07g0418600 mRNA	+3.9	+5.6	+5.5
Os07g0457800	Os07g0457800 mRNA	+4.6	+5.0	+8.7
Os07g0468200	Os07g0468200 mRNA	+3.1	+3.0	+4.4
Os07g0678400	Os07g0678400 mRNA	-3.1	-3.1	-3.2
LOC_Os08g32930	expressed protein	+3.3	+4.2	+5.1
LOC_Os08g31860	expressed protein	+3.4	+3.0	+3.2
LOC_Os09g02180	expressed protein	+3.5	+2.5	+4.4
LOC_Os03g04080	expressed protein	+2.8	+2.1	+4.5

LOC_Os06g38660	expressed protein	+3.2	+3.9	+3.9
LOC_Os09g23590	expressed protein	+3.3	+2.4	+4.6
Os09g0513500	Os09g0513500 mRNA	+4.9	+3.6	+3.2
Os01g0124400	Os01g0124400 mRNA	+4.6	+3.2	+5.9
Os01g0695800	Os01g0695800 mRNA	+3.1	+4.3	+3.6
Os01g0944900	Os01g0944900 mRNA	+4.8	+3.3	+4.6
Os02g0155400	Os02g0155400 mRNA	+2.6	+3.6	+3.8
Os07g0418700	Os07g0418700 mRNA	+5.8	+9.3	+7.9
Os10g0515900	Os10g0515900 mRNA	+5.6	+11.2	+6.0
Os12g0562000	Os12g0562000 mRNA	+4.8	+3.9	+6.5
Os02g0494300	Os02g0494300 mRNA	+7.0	+5.1	+9.9
Os03g0120600	Os03g0120600 mRNA	+9.8	+11.1	+12.1
Os04g0600200	Os04g0600200 mRNA	+2.7	+2.8	+3.7
LOC_Os10g23240	hypothetical protein	+11.7	+9.0	+15.1
LOC_Os12g11550	hypothetical protein	+4.5	+3.5	+4.4
LOC_Os12g11620	hypothetical protein	+4.4	+6.0	+5.2
LOC_Os12g17804	hypothetical protein	+3.0	+2.0	+3.3
LOC_Os07g43604	hypothetical protein	+3.2	+1.9	+3.3

Differentially abundant spots with a fold change ≥1.5 at least in one of the tested circumstances were considered. Differentially expressed transcripts in the three situations and with a fold change ≥3 at least in one of the tested conditions were considered. Highlighted in **red** are the situations where similar identifications were found in proteomics and microarrays. Green means that the identification corresponds to a protein which is "non-allergen" as predicted by AlgPred software hybrid approach. Orange represents "allergen" proteins as predicted by Algpred hybrid approach.
